# Supplementary material for: Ligand Binding Site Structure Influences the Evolution of Protein Complex Function and Topology
Source: Cell Rep. 2018 Mar 20;22(12):3265–76. doi: 10.1016/j.celrep.2018.02.085 (PMC5873459; doi:10.1016/j.celrep.2018.02.085)
Supplement: Document S1. Figures S1–S7 [file mmc1.pdf]

Cell Reports, Volume *22*

Supplemental Information

Ligand Binding Site Structure Influences the Evolution of Protein Complex Function and Topology

György Abrusán and Joseph A. Marsh

SUPPLEMENTARY FIGURES

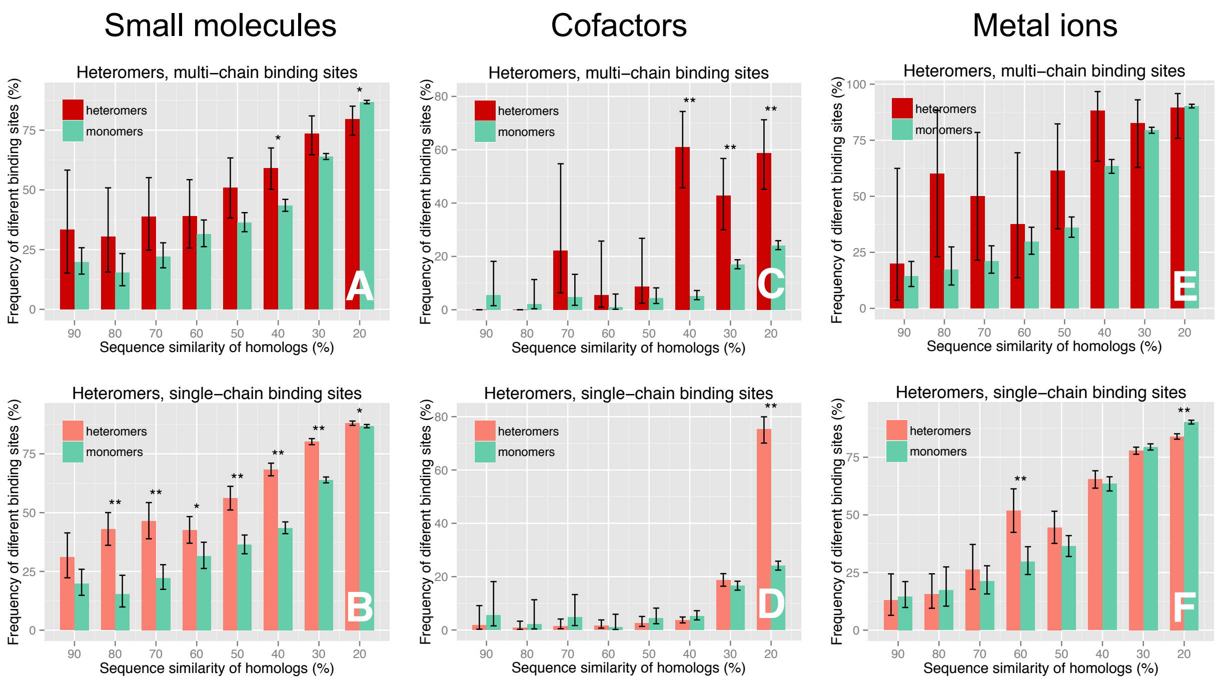

Figure S1, Related to Figure2. Evolution of ligand binding of small organic ligands (first column, panels **A**,**B**), cofactors (second column, panels **C**,**D**) and metal ions (third column, panels **E**,**F**) of heteromers. Whiskers represent 95% confidence intervals, whiskers for bins with zero frequency were not drawn; **: $p < 0.005$, *: $p < 0.05$; tests of proportions, with Benjamini-Hochberg correction for multiple testing.

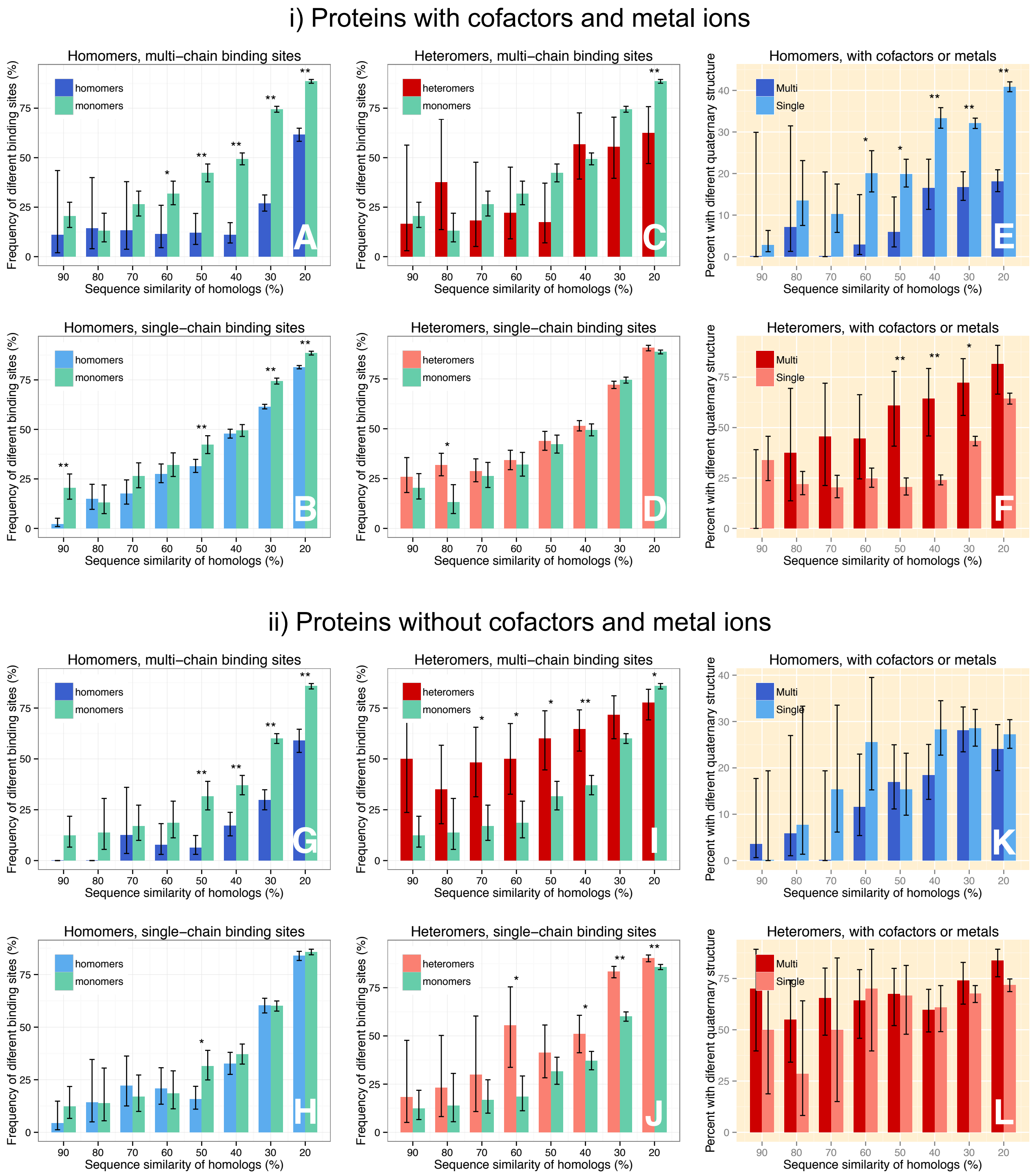

Figure S2, Related to Figure 2. Evolution of ligand binding and quaternary structure in proteins that bind metals or cofactors (panels **A-F**) and in proteins that do not (panels **G-L**). In the case of proteins with cofactors the conservation quaternary structure (unit number) of MBS and SBS complexes is markedly different for both homomers and heteromers (**E,F**), but show opposite patterns, while in the case of proteins without cofactors no significant difference exists (**K,L**). In the case of heteromers without cofactors (**L**) the lack of a positive correlation between sequence divergence and quaternary structure change suggests that the pattern is largely influenced by the incompleteness of structures in PDB. (Whiskers represent 95% confidence intervals, whiskers for bins with zero frequency wee not drawn; **: $p < 0.005$, *: $p < 0.05$; tests of proportions, with Benjamini-Hochberg correction for multiple testing.)

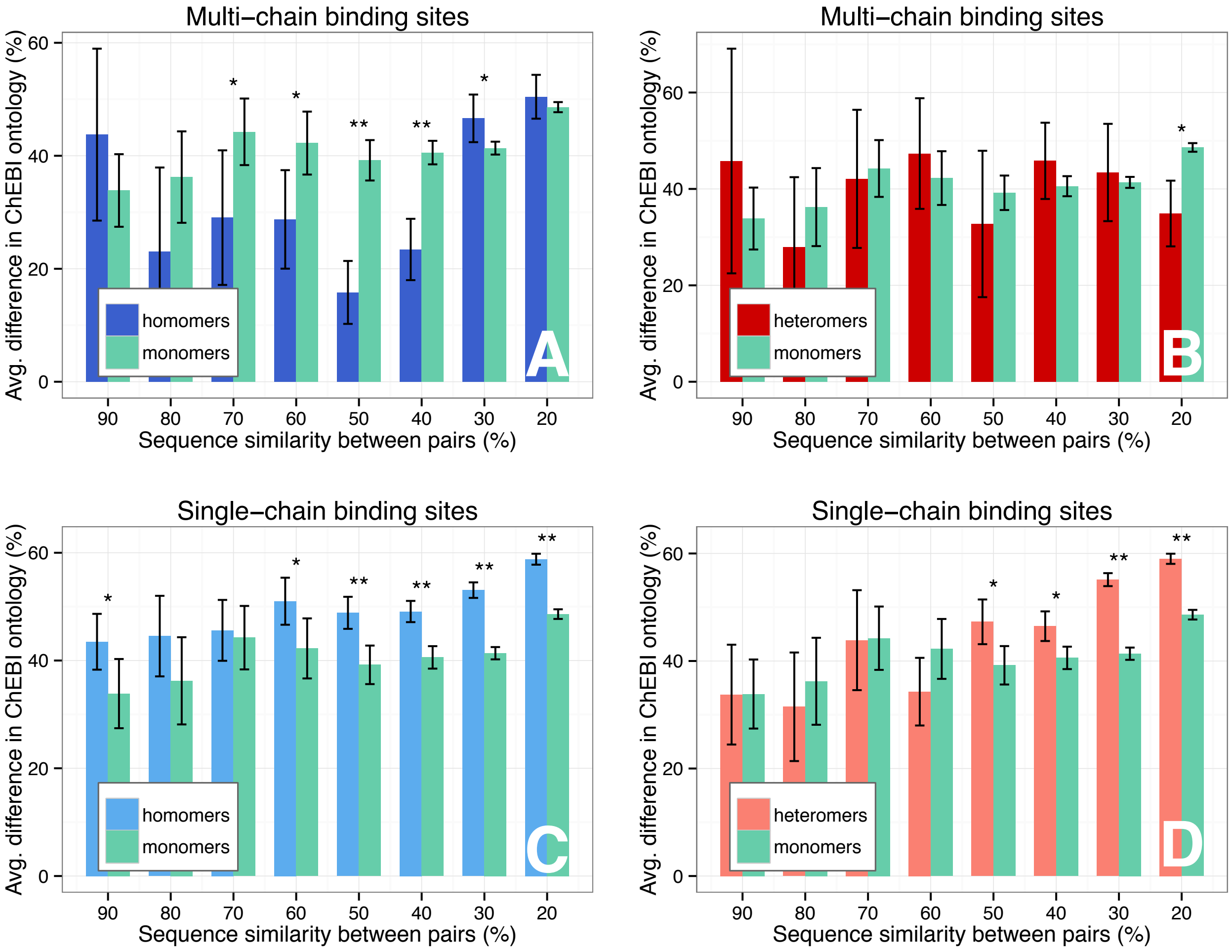

Figure S3. The change in chemical similarity of ligands, using only small molecules. Related to Figure 3. The pattern is qualitatively similar to the pattern shown on Figure 3, which includes also cofactors.

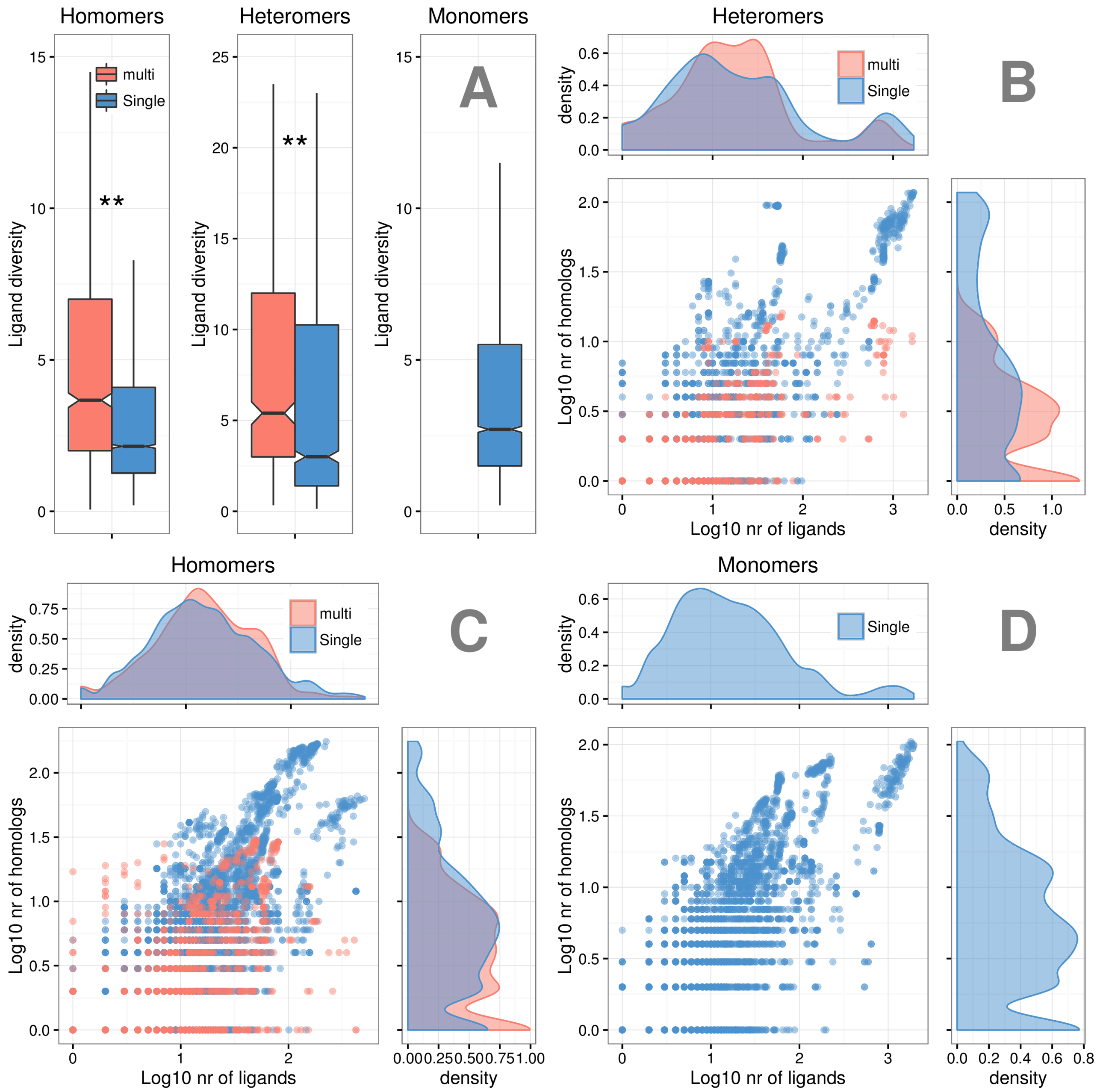

Figure S4. Complexes with multi-chain binding sites have more diverse ligand sets. Related to Figures 3 and 4. **A)** Ligand diversity (number of different ligands / number of homologous sequences) of different complex types. Note that the magnitude of the structural difference between different ligands was ignored, thus the differences quantify research effort, and not structural variability of ligands (for example, ATP and ADP are different ligands, although they are structurally very similar). Proteins with multi-chain binding sites have more ligands per sequence, and the difference is most pronounced in the case of homomers (**: $p < 0.005$, Wilcoxon tests). This indicates that the observed lower structural variability of ligands of homomers with multichain binding sites is not the consequence of systematic biases in ligand numbers, or the number of homologs in the PDB. Note that it is difficult to compare diversity between homomers and heteromers, because the latter have a much more variable sequence composition, resulting in higher overall ligand diversity. **B-D)** Variability in the number of homologous sequences and the number of different ligands of homomers, heteromers and monomers.

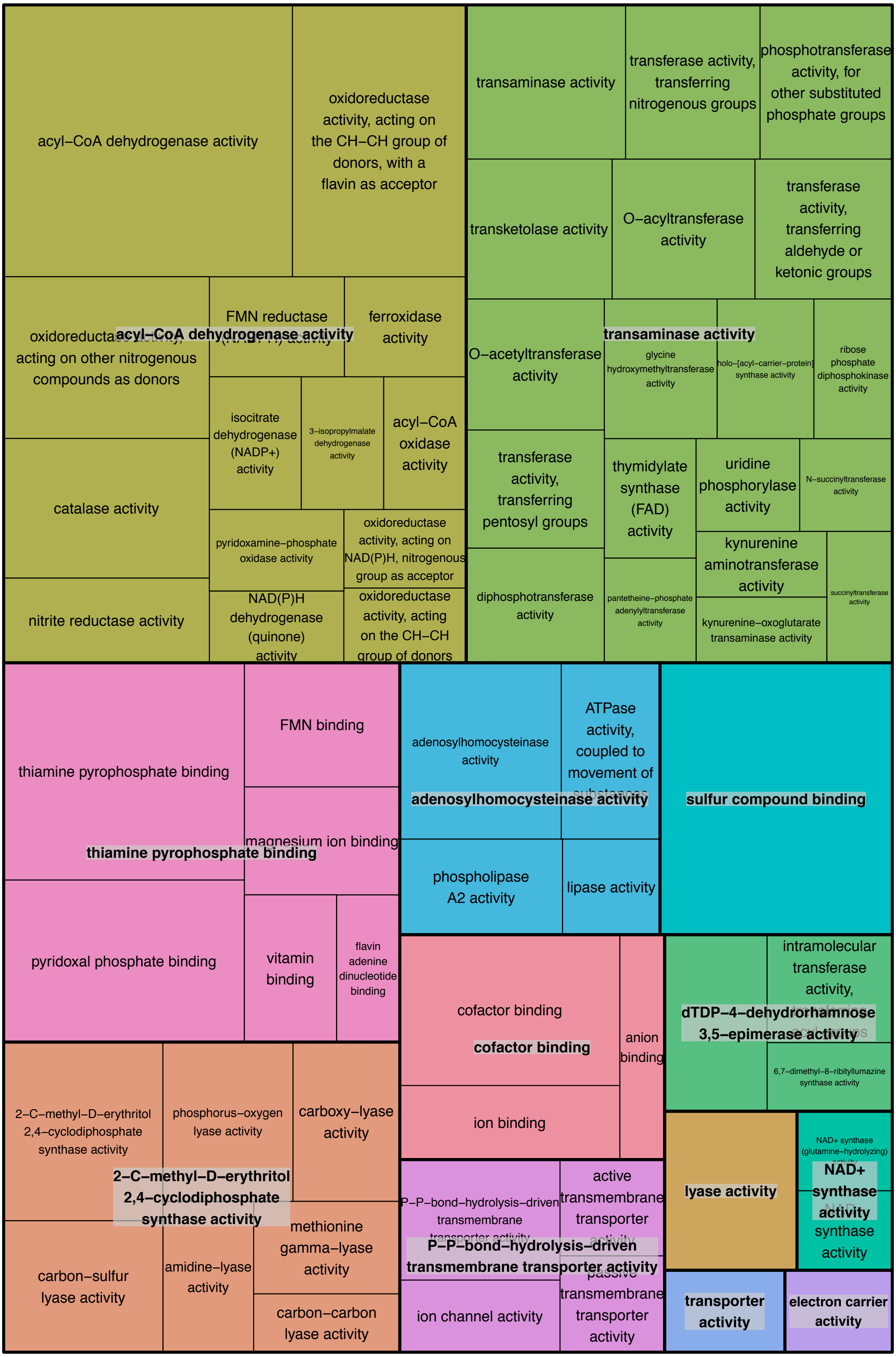
acyl−CoA dehydrogenase activity
oxidoreductase activity, acting on the CH−CH group of donors, with a flavin as acceptor
acting on other nitrogenous compounds as donors
acyl−CoA dehydrogenase activity
FMN reductase
ferroxidase activity
isocitrate dehydrogenase (NADP+) activity
3−isopropylmalate dehydrogenase activity
acyl−CoA oxidase activity
catalase activity
pyridoxamine−phosphate oxidase activity
oxidoreductase activity, acting on NAD(P)H, nitrogenous group as acceptor
nitrite reductase activity
NAD(P)H dehydrogenase (quinone) activity
oxidoreductase activity, acting on the CH−CH group of donors
transaminase activity
transferase activity, transferring nitrogenous groups
phosphotransferase activity, for other substituted phosphate groups
transketolase activity
O−acyltransferase activity
transferase activity, transferring aldehyde or ketonic groups
O−acetyltransferase activity
transaminase activity
glycine hydroxymethyltransferase activity
holo−[acyl−carrier−protein] synthase activity
ribose phosphate diphosphokinase activity
transferase activity, transferring pentosyl groups
thymidylate synthase (FAD) activity
uridine phosphorylase activity
N−succinyltransferase activity
kynurenine aminotransferase activity
diphosphotransferase activity
pantetheine−phosphate adenylyltransferase activity
kynurenine−oxoglutarate transaminase activity
succinyltransferase activity
thiamine pyrophosphate binding
FMN binding
magnesium ion binding
thiamine pyrophosphate binding
pyridoxal phosphate binding
vitamin binding
flavin adenine dinucleotide binding
adenosylhomocysteinase activity
ATPase activity, coupled to movement of
adenosylhomocysteinase activity
phospholipase A2 activity
lipase activity
sulfur compound binding
cofactor binding
cofactor binding
anion binding
ion binding
intramolecular transferase activity,
dTDP−4−dehydrorhamnose 3,5−epimerase activity
6,7−dimethyl−8−ribityllumazine synthase activity
2−C−methyl−D−erythritol 2,4−cyclodiphosphate synthase activity
phosphorus−oxygen lyase activity
carboxy−lyase activity
2−C−methyl−D−erythritol 2,4−cyclodiphosphate synthase activity
methionine gamma−lyase activity
carbon−sulfur lyase activity
amidine−lyase activity
carbon−carbon lyase activity
P−P−bond−hydrolysis−driven transmembrane
active transmembrane transporter
P−P−bond−hydrolysis−driven transmembrane transporter activity
ion channel activity
transmembrane transporter activity
lyase activity
NAD+ synthase (glutamine−hydrolyzing)
NAD+ synthase activity
synthase activity
transporter activity
electron carrier activity

Figure S5 (previous page). TreeMap of significantly overrepresented GO terms (molecular function) of MBS homomers. Related to Figure 5. The size of rectangles is proportional to the p-value of the terms, colors indicate clusters of related terms. See also Table S1.

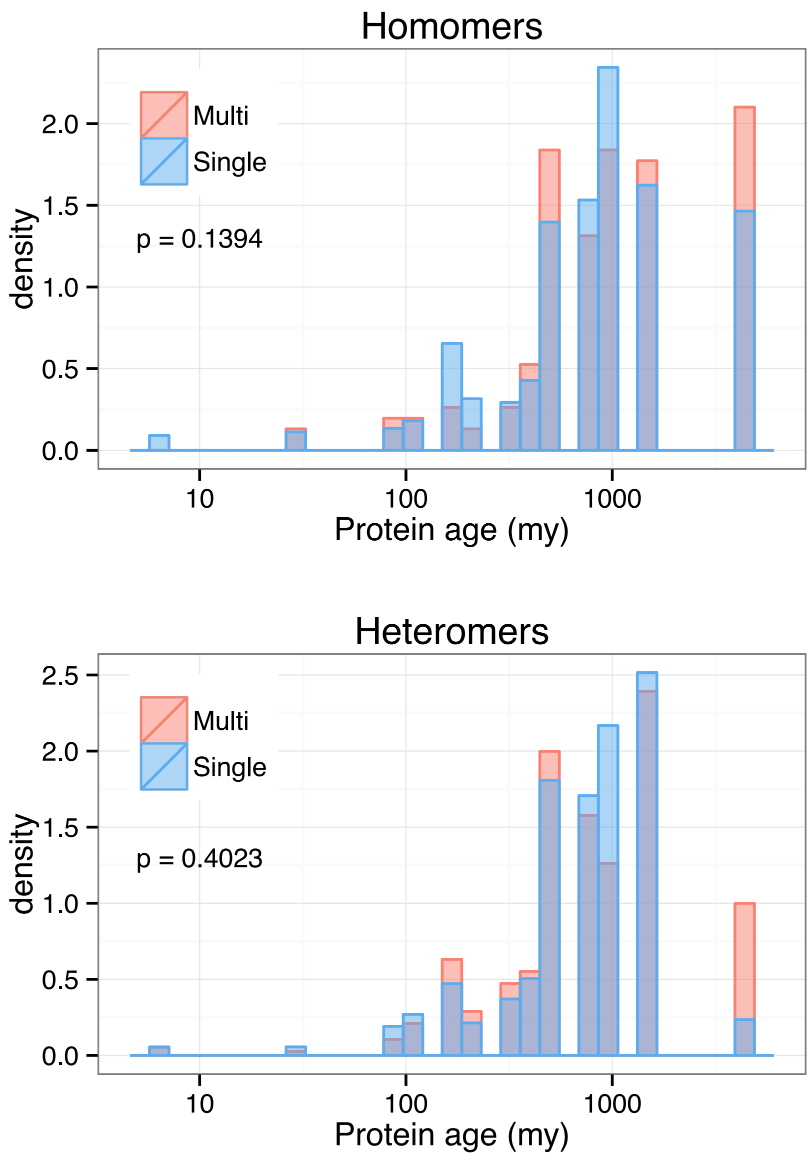

Figure S6, related to Figure 6. The age of proteins of complexes with MBS binding sites is not significantly different from the proteins of SBS complexes, although the difference between MBS and SBS proteins is largest in the oldest bin, representing proteins that were already present in the last universal common ancestor (LUCA) of all cellular organisms. Note that several complexes contain both types of binding sites, these are present in both distributions. Significance was tested with two sample Wilcoxon tests.

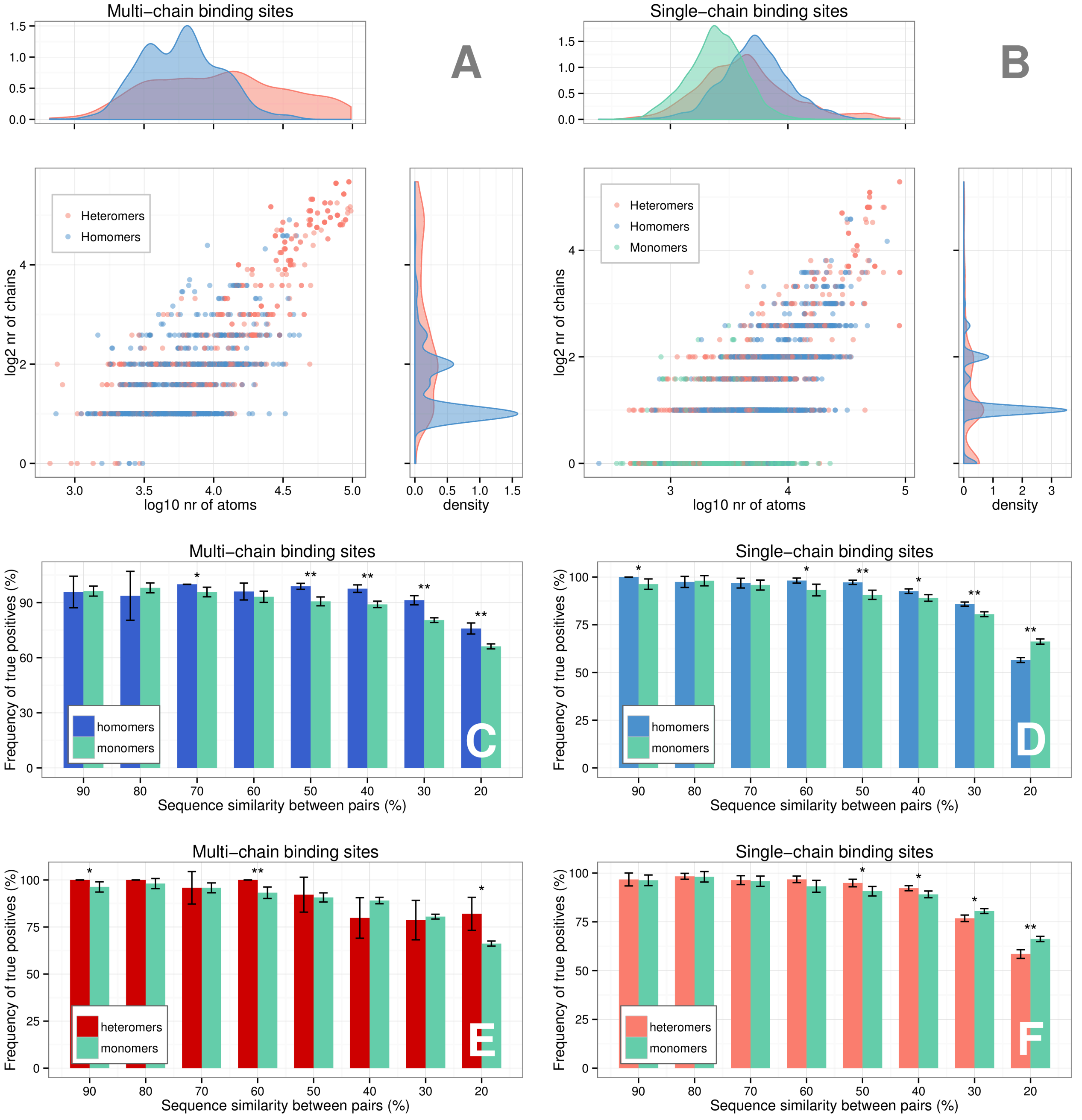

Figure S7, related to Figure 2. The efficiency of binding pocket detection is not worse in complexes than in monomers. Efficiency was determined as the fraction of significant hits between homologous structures that are known to bind the same ligand ("true positives"). **A-B)** Homomers and heteromers can have much higher numbers of chains and atoms than monomers, both in the case of complexes with multi- and single-chain binding sites. **C-F)** Despite the larger structures of complexes, the detection rate of pockets with similar ligands is comparably high in complexes and monomers, and in the case of homomers with multi-chain binding sites it is even higher than in monomers (whiskers represent 95% confidence intervals, **: $p < 0.005$, *: $p < 0.05$, t-tests, with Benjamini-Hochberg correction).
