# Supplementary material for: Ligand Binding Site Structure Influences the Evolution of Protein Complex Function and Topology
Source: Cell Rep. 2018 Mar 20;22(12):3265–76. doi: 10.1016/j.celrep.2018.02.085 (PMC5873459; doi:10.1016/j.celrep.2018.02.085)
Supplement: Document S2. Article plus Supplemental Information [file mmc6.pdf]

Cell Reports

Article

Ligand Binding Site Structure Influences the Evolution of Protein Complex Function and Topology

Graphical Abstract

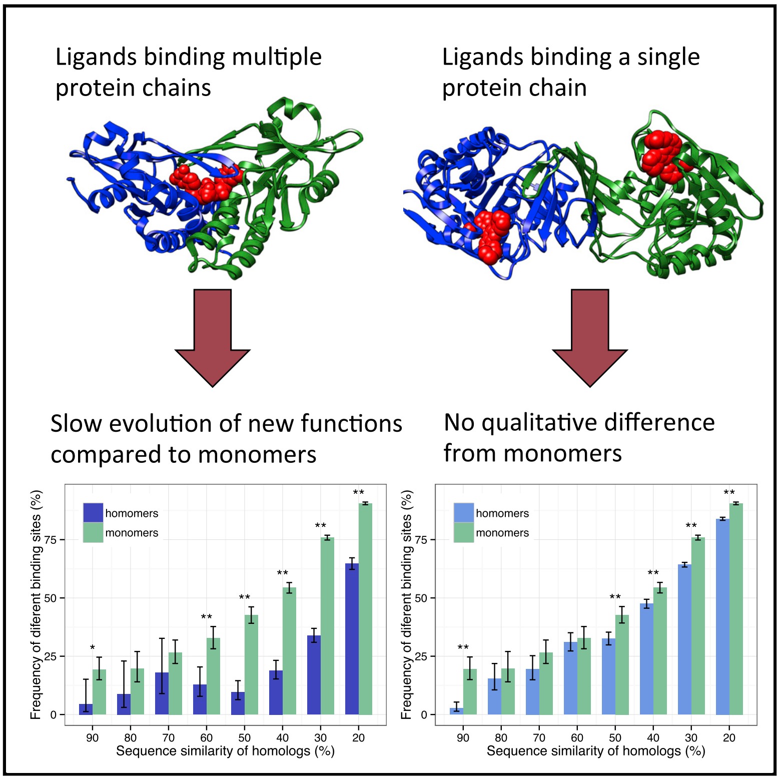

Authors

György Abrusán, Joseph A. Marsh

Correspondence

gyorgy.abrusan@igmm.ed.ac.uk

In Brief

Homomers with ligand binding sites involving multiple protein chains (MBS homomers) evolve new functions slower than other homomers and monomers, and the ones binding cofactors/metals also have more conserved quaternary structure (QS). These complexes are likely to be promising targets for antibiotics and multitarget drugs.

Highlights

- Ligand binding site structure significantly influences protein function evolution
- MBS homomers have more similar ligand binding pockets than monomers and other homomers
- Cofactor and metal-binding MBS homomers have more conserved QS than other homomers
- MBS homomers are promising targets for developing antibiotics and multitarget drugs

Abrusán & Marsh, 2018, Cell Reports *22*, 3265–3276
March 20, 2018 © 2018 The Author(s).
https://doi.org/10.1016/j.celrep.2018.02.085

Cell Reports
Article

OPEN ACCESS
CellPress

Ligand Binding Site Structure Influences the Evolution of Protein Complex Function and Topology

György Abrusán[1,2,*] and Joseph A. Marsh[1]
[1]MRC Human Genetics Unit, Institute of Genetics and Molecular Medicine, University of Edinburgh, Crewe Road, Edinburgh EH4 2XU, UK
[2]Lead Contact
*Correspondence: gyorgy.abrusan@igmm.ed.ac.uk
https://doi.org/10.1016/j.celrep.2018.02.085

SUMMARY

It has been suggested that the evolution of protein complexes is significantly influenced by stochastic, non-adaptive processes. Using ligand binding as a proxy of function, we show that the structure of ligand-binding sites significantly influences the evolution of protein complexes. We show that homomers with multi-chain binding sites (MBSs) evolve new functions slower than monomers or other homomers, and those binding cofactors and metals have more conserved quaternary structure than other homomers. Moreover, the ligands and ligand-binding pockets of homologous MBS homomers are more similar than monomers and other homomers. Our results suggest strong evolutionary selection for quaternary structure in cofactor-binding MBS homomers, whereas neutral processes are more important in complexes with single-chain binding sites. They also have pharmacological implications, suggesting that complexes with single-chain binding sites are better targets for selective drugs, whereas MBS homomers are good candidates for broad-spectrum antibiotic and multitarget drug design.

INTRODUCTION

The majority of genomic traits show dramatic changes with organismal complexity and genome size (Lynch, 2007). Due to their high effective population size, prokaryotes are characterized by small, dense genomes, where the amount of coding sequence typically takes up more than 90% of the genome. In contrast, in higher eukaryotes, the fraction of coding sequence takes up only a small fraction (1%–2%) of the genome, and its fraction changes gradually with genome size. A similar trend is present for many other traits: intron size, intron number, number of genomic parasites, codon bias, and recombination rate all show clear correlations with the size of the genome (Lynch and Conery, 2003), which is caused by the very different effective population sizes and, in consequence, strength of selection in species with large and small genomes (see Lynch, 2007 for an overview).

There is, however, a notable exception to this pattern: protein complexes. The majority of proteins do not function in isolation but form complexes, which can be either homomeric, i.e., are formed by several units of the same protein, or heteromeric, which are formed by several different proteins (see recent reviews by Levy and Teichmann, 2013 and Marsh and Teichmann, 2015). Whereas it is known that, during crystallization, non-physiological complexes are formed frequently, an implicit assumption of most studies on complex evolution is that the structure of protein complexes is adaptive and is a result of selection (Ali and Imperiali, 2005; Marianayagam et al., 2004; Nishi et al., 2013). This assumption is highly intuitive and is supported by numerous observations. For example, pathogenic mutations are highly enriched in protein interfaces (Sahni et al., 2015; Yates and Sternberg, 2013), quaternary structure is frequently conserved (Levy et al., 2008), protein complex assembly pathways tend to be evolutionarily optimized and conserved (Marsh et al., 2013; Wells et al., 2016), and different biological functions are strongly associated with different types of quaternary structure (Bergendahl and Marsh, 2017). However, Lynch (2013) has recently pointed out that, unlike most genomic traits, the frequency of protein complexes in different taxa, and their quaternary structure, do not show the same dramatic changes with the complexity of organisms (and in consequence, their effective population size) as most genomic traits and thus do not scale with the strength of selection. This suggests that stochastic processes may play a fundamental role in the evolution of complexes, particularly in the case of homomers (Lynch, 2013). Further support for this hypothesis comes from the observation that the global distribution of quaternary structure topologies in complexes with experimentally determined structures is largely consistent with the distribution expected from random combinations of evolutionary steps (Ahnert et al., 2015).

In this paper, using ligand binding characteristics of proteins present in the PDB, we test whether the acquisition of novel functions/ligands depends on protein quaternary structure and whether it depends on the structure of the ligand binding pocket. We hypothesized that changes in the assembly and organization of protein complexes can result in a rapid emergence of novel functions without dramatic changes in the tertiary structure if the functional sites are formed by more than one protein in the complex (thus changes in assembly might be a source of evolutionary innovations). Whereas protein-ligand interactions have been studied for decades and are of fundamental importance

This is an open access article under the CC BY license (http://creativecommons.org/licenses/by/4.0/).

OPEN ACCESS
CellPress

in drug discovery (where it is generally assumed that similar binding sites bind similar ligands; Klabunde, 2007), large, PDB-scale analyses of protein-ligand interactions have so far characterized such interactions only at a protein domain or fold level (Furnham et al., 2016; Ji et al., 2007; Kinjo and Nakamura, 2009; Nath et al., 2014), and we are not aware of any study that tested whether there are general relationships between complex formation, complex evolution, and ligand binding. We find that the structure of the ligand binding pocket has a profound influence on the evolution of protein function. In the case of complexes where the residues of a binding site are restricted to a single protein chain, the characteristics of ligand binding in protein complexes and monomers do not differ qualitatively, suggesting that, in the case of these complexes, the "Lynch conjecture" is likely to be correct and neutral processes are very important in the evolution of quaternary structure of such complexes. In contrast, in homomers with small-molecule binding sites involving residues from several protein chains, we observe clear differences that contradict our initial hypothesis: they acquire novel ligands/functions much slower than monomers and bind chemically more similar ligands. In addition, in the case of homomers with multi-chain binding metals or cofactors, we observe a markedly lower variability in quaternary structure (unit number) than in homomers with single-chain binding sites (SBSs). We also find that the ligands of homomers with multi-chain binding sites (MBSs) show significantly lower structural variability than monomers or other homomers, are significantly enriched in nucleobase-containing ligands, and frequently perform metabolic functions.

RESULTS

The Structure of Ligand Binding Site Influences the Evolution of Function of Complexes

We use ligand binding and the presence of binding pockets as a proxy of protein function because it allows comparisons on a PDB-wide scale and is based on experimental data; thus, one can compare homologs without the assumption of functional similarity. To test whether the evolution of protein function is significantly influenced by quaternary structure, we used the BioLiP database (Yang et al., 2013), a semi-manually curated database of protein-ligand interactions. BioLiP contains only ligands that are likely to be biologically relevant (and thus are not artifacts of crystallization, such as solvents used in structure determination) and also provides an annotation of the ligand binding residues. We classified homomer and heteromer binding sites into two groups: sites where the binding residues are located on only one chain of the complex (SBSs) and sites where the binding residues of a particular ligand are located on more than one chain (MBSs; Figures 1A and 1B). We grouped ligands into three categories: cofactors; metals and small molecules; and excluded nucleic acids and peptide ligands from the analysis. The distribution of the three ligand types is not identical in the five protein groups. Whereas small molecules dominate the ligands in all groups, MBS complexes are characterized with a higher frequency of cofactors and fewer metals (Figure 1C) and have somewhat fewer ligands per protein than SBS complexes and monomers (Figure 1D).

We examined the evolution of protein function by testing how the ability of binding the ligands of homologous structures changes with their sequence similarity. We used ProBiS (Konc and Janežič, 2010, 2017) to search for the presence of ligand binding sites in homologous proteins (Experimental Procedures and Figure 1E) and ordered the frequency of different binding sites (i.e., the frequency of sites not having a significant hit in a homolog) according to the sequence similarity of homologous sequence pairs (Figure 2). The comparison of homomers and heteromers with monomers indicates that the binding sites of homomers with MBSs are much more likely to be found in homologs than in SBSs or monomers (Figures 2A and 2B), particularly in the case of distant homologs. In contrast, heteromers with multi-chain binding pockets do not show the same pattern (Figures 2C and 2D).

The higher conservation of MBSs of homomers (Figure 2A) is caused by two independent processes: the significantly slower divergence of small-molecule binding pockets and the somewhat different frequencies of cofactors and metals in this protein group. The three ligand categories show different evolutionary patterns: the binding pockets of small molecules show much higher conservation in MBS homomers than in monomers or SBS homomers (Figures 2E and 2F), whereas, in case of heteromers, their binding sites appear to diverge faster than in monomers (Figures S1A and S1B). The binding pockets of cofactors are much more conserved: almost no differences are present above 40% sequence similarity (Figures 2G, 2H, S1C, and S1D), which is caused by their ancient evolutionary origins, possibly dating back to the RNA world (reviewed in Smith and Morowitz, 2016) but also by the very low structural diversity of this group, i.e., the fact that many distantly related homologs share the same cofactor. In contrast, the binding pockets of metal ions are the less conserved and show no clear differences between the five protein groups (Figures 2I, 2J, S1E, and S1F), which may be partly caused by the small size of these pockets, resulting in lower efficiency of detection in homologous proteins.

Cofactors and Metals Binding Multiple Protein Chains Influence the Evolution of Quaternary Structure

Next, we tested whether the observed differences in binding result in differences in the variability of quaternary structure, as defined by the number of subunits in a complex. We found that, in the case of proteins with MBS cofactors or metals, quaternary structure evolves differently in MBS and SBS complexes (Figures 2K, 2L, S2E, and S2F), but surprisingly, in MBS complexes binding only small molecules, there is no such effect (Figures S2K and S2L). In homomers with codactors/metals, MBS complexes show a much higher conservation of unit number than in SBS complexes (Figures 2K and S2), whereas in heteromers, the pattern is the opposite: the structure of such MBS heteromers appear to change faster than SBS heteromers (Figures 2L and S2F). Besides contributing to catalysis (Fischer et al., 2010), certain cofactors and metals are known to stabilize the tertiary structure of proteins; classic examples include the heme of myoglobin and cytochrome-c. Our results strongly suggest that MBS cofactors and metals also influence the formation and evolution of protein complexes. In MBS homomers, their effect is most likely stabilizing, resulting in slower

OPEN
ACCESS
CellPress

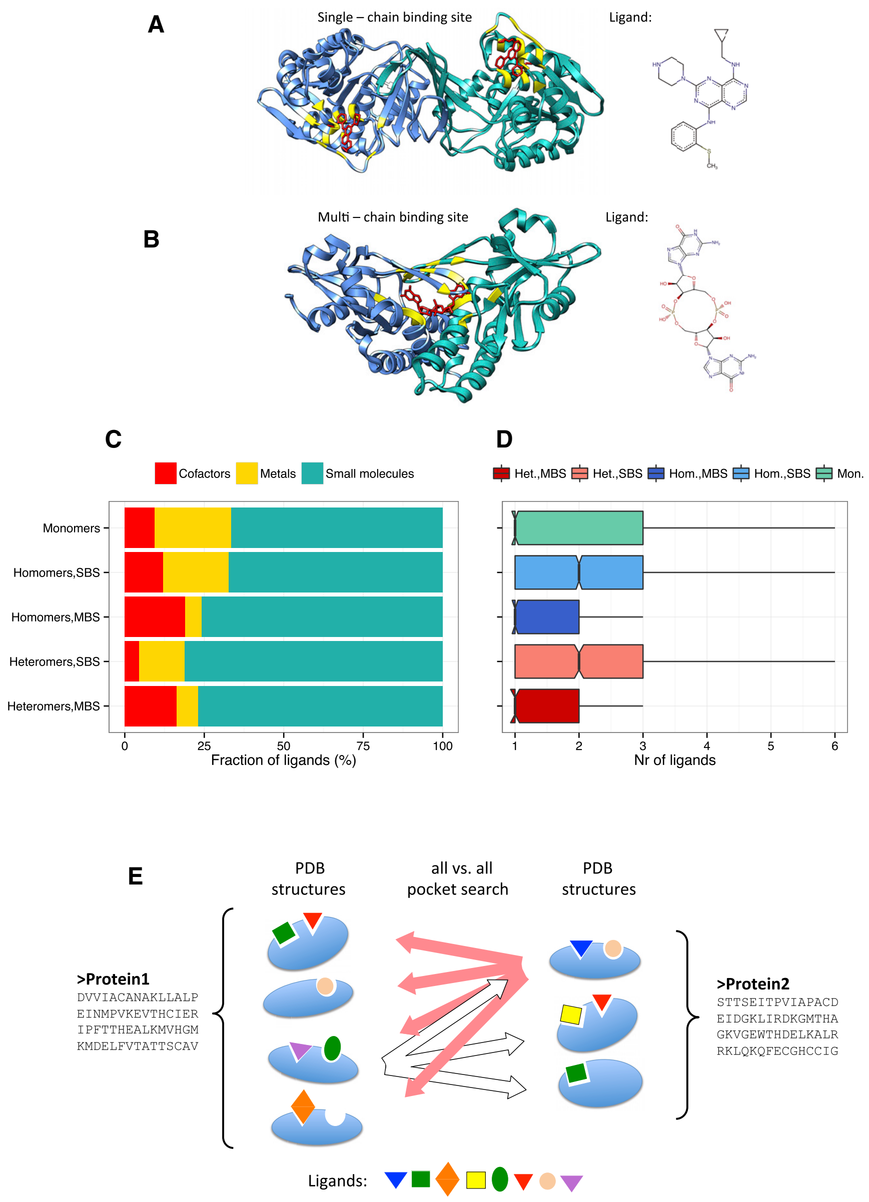

Figure 1. Examples of SBSs and MBSs in Homomers and Ligand Distributions

(A) Human ketohexokinase complexed with pyrimidopyrimidine (PDB ID: 3Q92). The protein forms a dimer, and each subunit has an independent binding pocket, containing a ligand (red). Residues binding the ligand are highlighted in yellow.

(B) Anemone STING (stimulator of interferon [IFN] genes) protein, complexed with cyclic diguanosine monophosphate (PDB ID: 5CFL). The homodimer has only one binding site, which is formed by both protein chains. Residues binding the ligand are highlighted in yellow.

(C) The composition of biologically relevant ligands in monomers, SBS homomers, MBS homomers, SBS heteromers, and MBS heteromers. In all cases, the majority of ligands are small organic molecules, but MBS complexes are characterized with higher fraction of cofactors and fewer metal ions. Nucleic acids and peptides were not used in the analyses.

(D) Boxplots of the number of ligands in the five protein categories, excluding outlayers. The number of different ligands per protein follows an exponential-like distribution, with the majority of proteins having one or two different ligands.

(E) Outline of the ligand binding pocket searches: for each pair of homologous proteins, we performed an exhaustive search, i.e., we searched all structures of the target protein with all ligand binding pockets of all structures of the query protein, using both proteins as target and query.

evolution of quaternary structure, and selection is likely to influence the assembly and composition of such homomers more than of SBS homomers. In contrast, in MBS heteromers, they seem to contribute to evolutionary innovations (Figure 2L).

In the case of complexes without cofactors or metals, the evolution of quaternary structure does not seem to evolve differently in SBS and MBS complexes, despite the clear differences in their ligand binding patterns (Figures S2G–S2L). However, in the case of such heteromers, the lack of increasing trend with sequence divergence (i.e., the fact the more distant sequences do not have more different quaternary structure; Figure S2L) suggest that, besides biological factors, the observed patterns are also significantly influenced by biases in the PDB. This could be caused by incomplete crystals or the recent availability of cryoelectron microscopy (cryoEM) structures, which are typically much larger than the structures obtained with X-ray crystallography.

Complexes with MBSs Have Chemically Less Variable Ligands and Binding Sites

What mechanism could be responsible for the slower functional change seen in MBS homomers? There are two fundamentally different possibilities. First, MBSs may be more generic and flexible, and in consequence, they may be able to process a broader spectrum of ligands. Examples of this are certain membrane proteins, receptors, and regulatory proteins (Pabon and Camacho, 2017). It has been shown recently that, in the case of a multidrug resistance protein, the conformational variability of its bipartite binding site is the most likely cause of its very broad substrate specificity (Johnson and Chen, 2017). However, flexible binding sites and broad substrate specificity are also present in some SBS homomers (Hvorecny et al., 2017) and even monomers (Fong et al., 2017). Alternatively, the functions and ligands of MBS homomers may be more conserved than of SBS homomers.

We tested which of these hypotheses is true by comparing the chemical characteristics of ligands and the structural

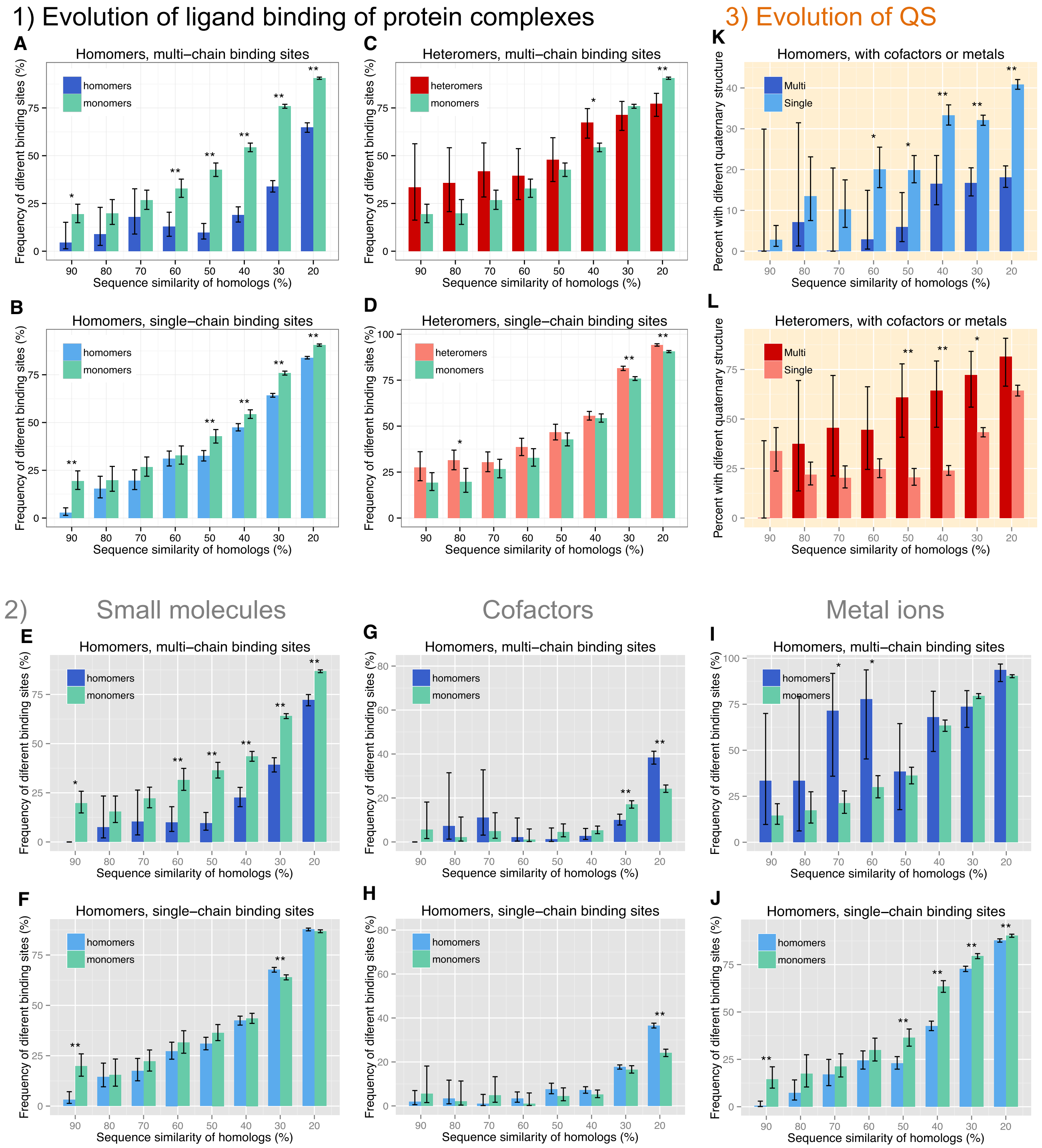

Figure 2. Evolution of Ligand Binding and QS

We use the ability to bind ligands of homologous proteins (scaled with sequence similarity) as the measure of their functional similarity.

(1) General relationships between ligand binding and QS in homomers, heteromers, and monomers.

(A) Homomers with MBSs show a much slower functional divergence than monomers. This pattern is caused by two separate processes: (1) the binding pockets of small-molecule ligands diverge significantly less rapidly than the pockets of monomers and (2) MBS homomers have a higher fraction of cofactors, which are frequently identical and have highly conserved binding pockets plus fewer metal ions.

(B) In homomers with binding sites restricted to a single chain, the ability to bind the ligands of homologs changes qualitatively similarly to monomers.

(C) MBS heteromers do not show the same pattern as MBS homomers.

(legend continued on next page)

variability of the binding sites. We tested the chemical similarity of ligands of homologous structures using the ChEBI (Chemical Entities of Biological Interest) ontology database (Degtyarenko et al., 2008; Hastings et al., 2016). ChEBI provides a manually curated hierarchical ontology, conceptually similar to gene ontology (GO), that is based on chemical structure (Figure 3A). We used the number of different terms between chemical compounds in the ontology to quantify the chemical and structural difference between organic ligands, excluding nucleic acids, and peptides (Figure 3A). Our analysis shows that, in the case of MBS homomers, the difference between ligands of homologs is smaller than between ligands of monomers, whereas in the case of SBS complexes, the difference is somewhat larger or not significant (Figures 3B–3E). Excluding cofactors from the analyzed ligands does not change the pattern dramatically (Figure S3), although it does influence the most diverged homologs.

Ligand binding frequently results in conformational changes of proteins, and we also tested the structural variability of the binding site by comparing the root mean square deviation (RMSD) of the significant hits (with Z score > 2) of homologs with sequence similarity between 30% and 49%. Because RMSD depends on the size of the superposed structures, we used the size of the matching pocket as a covariate. We expected that hits to binding sites in proteins with more different ligands and binding sites will have higher RMSD. As predicted, we found a significant difference between MBS and SBS homomers (Figure 4A), but we found no consistent difference in heteromers (Figure 4B), irrespectively whether cofactors were included or excluded from the dataset. Additionally, using those proteins where ligand binding (holo) and ligand-free (apo) structures are both present in the PDB, we tested whether ligand binding results in a larger conformational change in MBS homomers than SBS homomers (measured as the RMSD of the ligand binding pocket of the holo structure in the apo structure). We did not find a clear difference between the two (Figure 4D). Our results based on pocket comparison support the findings based on chemical similarity: on average, the structural variability of the binding sites in MBS homomers is lower than of SBS homomers, and the cases where flexibility is assumed to result in ligand promiscuity are most likely a minority.

The PDB is not an unbiased database, as its composition is significantly influenced by the priorities of the research community. Thus, the observed differences could also be caused by biases in the PDB rather than real biological differences, if MBS homomers have consistently more homologs in the PDB having identical ligands or have been systematically crystallized with fewer ligands. To rule this out, we determined the ligand diversity of sequences in the PDB, measured as the total number of ligands of the homologs of a sequence divided by the number of homologs it has (Figure S4). Note that, because we ignored structural differences between ligands, high diversity does not necessarily mean high structural variability (for example ATP and ADP are different ligands, although are structurally very similar) but simply the high number of non-identical compounds, irrespectively whether they are small modifications of each other or radically different; thus, this measures research effort and not structural variability. The results show that proteins with MBSs have higher number of ligands per sequence, indicating that the higher similarity of ligands and lower structural variability of MBS homomers are not caused by their lower ligand diversity in the PDB (Figure S4).

Functional Analysis of MBS Homomers

To test whether MBS homomers have characteristic functions, we performed a Gene Ontology enrichment analysis and also an enrichment analysis of the ChEBI terms of their ligands. We found that, whereas the molecular functions of MBS homomers are diverse, several functions are significantly enriched in comparison to all ligand binding homomers, including acyl-coenzyme A (CoA) dehydrogenase activity, transaminase activity, thiamine pyrophosphate binding, cofactor binding, transmembrane transport, ion binding, transporter activity, and other functions frequently involving cofactor binding (Figures 5A and S5; Table S1). Next, we tested whether the chemical composition of the ligands of MBS homomers is biased toward certain chemical groups by a ChEBI structural term enrichment analysis (Experimental Procedures). We found that the ligands that are present in ChEBI (of the almost 20,000 ligands present in BioLiP at the time of writing, only ~3,100 are present in ChEBI) show clear structural biases: the most enriched chemical structures are

(D) In SBS heteromers, similarly to SBS homomers, the ability to bind the ligands of homologs changes qualitatively similarly to monomers. In the case of SBS complexes, stochastic processes (i.e., drift) are likely to play a significant role in shaping the number of subunits or their topology.

(2) Evolution of ligand binding of different ligand categories in homomers (see Figure S1 *for heteromers*).

(E and F) The binding pockets of small molecules show much higher conservation in (E) MBS homomers than in monomers or (F) SBS homomers.

(G and H) In the case of cofactors, the binding pockets are highly conserved and show little difference between QS types (G, MBS homomers; H, SBS homomers).

(I and J) The metal binding pockets are the less conserved and show no qualitative differences between the groups, most likely due to their small size (I, MBS homomers; SBS homomers).

(3) Evolution of quaternary structure.

(K) In homomers that bind cofactors or metal ions, the structure of binding pockets have fundamental consequences for the evolution of quaternary structure (see also Figure S2). Homomers with multi-chain biding sites have significantly lower variability in quaternary structure than complexes with SBSs, suggesting that the structure of the binding pocket is an important determinant of the evolution of their quaternary structure.

(L) In the case of heteromers with cofactors and metals, we see the opposite pattern to homomers in the evolution of QS: MBS heteromers appear to change faster than SBS heteromers.

On all panels, bars represent proportions, whiskers 95% confidence intervals. **$p < 0.005$; *$p < 0.05$; tests of proportions, with Benjamini-Hochberg correction for multiple testing.

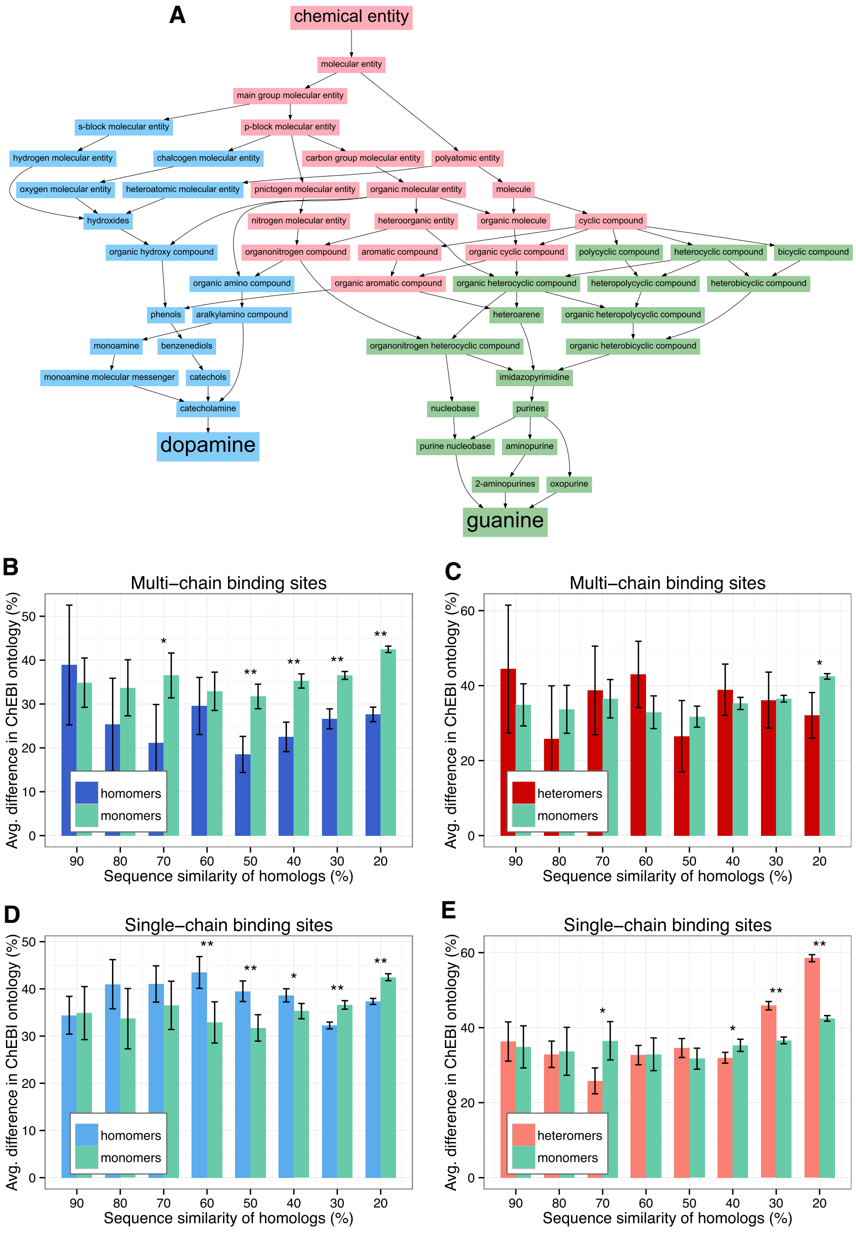

Figure 3. Homomers with MBSs Are Characterized with Chemically More Similar Ligands

(A) Example of ChEBI ontology similarity between two different chemical compounds (dopamine and guanine). Shared ontology terms are highlighted with red; dopamine and guanine specific terms are highlighted with blue and green, respectively. The difference in their chemical composition was measured as the proportion of the ligand specific and all terms, i.e., (blue + green)/all terms: (15 + 17)/ 49 = 65%.

(B and C) The chemical properties of ligands of homomers with MBSs (B) change less with sequence divergence than the properties of ligands of monomers or other complexes, but not in heteromers (C).

(D and E) Ligands of homomers with SBSs (D) show a weak but consistently higher variability in the chemical properties of ligands than monomers, while there is no clear difference between SBS heteromers (E) and monomers.

Bars represent averages; whiskers 95% confidence intervals; $^{**}p < 0.005$; $^{*}p < 0.05$; t tests, with Benjamini-Hochberg correction for multiple testing.

See also Figure S3.

nucleobase-containing ligands, frequently containing ribonucleotides (Figure 5B; Table S2). It has been long known that many protein cofactors are derivatives of nucleotides (Petsko and Ringe, 2008), and it has been suggested that this pattern is ancient and might have originated in the RNA world (Ji et al., 2007; White, 1976). However, excluding cofactors from the analyzed ligands does not change the pattern qualitatively. To assess the biochemical role of these ligands, we also performed an enrichment analysis of the ChEBI role ontology terms, which indicates that the most significantly enriched terms are metabolites, including fundamental metabolites (Figure 5C; Table S3), irrespectively whether cofactors are included or not. This suggests that MBS homomers are performing central, mostly metabolic, and probably ancient functions.

General Evolutionary and Structural Characteristics of Complexes with Single- and Multi-chain Binding Complexes

The patterns described above could also potentially emerge if MBS homomers are significantly younger and faster evolving than SBS monomers or homomers, which could result in the observed lower chemical variability, as younger protein families are expected to have less diverse functions (Osadchy and Kolodny, 2011). To test this, we compared the age of proteins that are part of MBS and SBS complexes, using the human protein age dataset provided by Capra et al. (2012, 2013; Experimental Procedures). We found no significant difference in the ages of MBS and SBS complexes (Figure S6). In fact, MBS complexes are somewhat more enriched in the oldest age groups, which is in agreement with the higher frequency of cofactor binding in these groups (Figure 1).

Ligand binding frequently results in conformational changes of both proteins and their ligands (Stockwell and Thornton, 2006), and flexibility has been shown to be essential for the proper functioning of ligand-binding proteins (Petsko and Ringe, 2008). Therefore, because differences in flexibility could have significant consequences for ligand binding, we compared the flexibilities of subunits from MBS and SBS complexes using a simple method based upon relative solvent

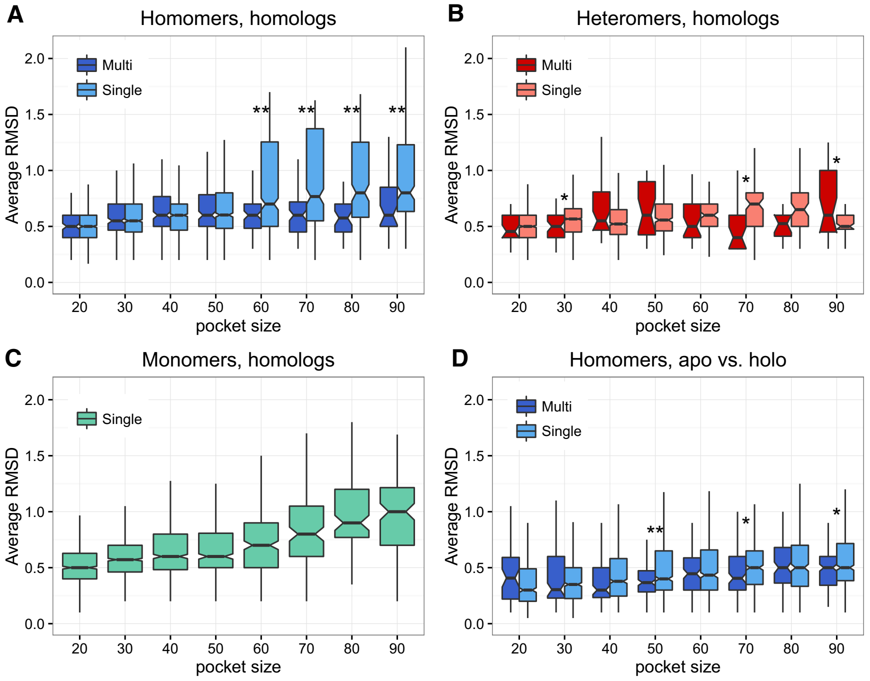

Figure 4. Homologs of MBS Homomers Have More Similar Binding Site Structure Than SBS Homomers or Monomers

(A–C) The comparison of binding sites of homologous proteins with 30%–50% sequence identity indicates that the structural variability of binding sites of (A) homomers (measured as the root mean square deviation [RMSD], in angstrom) with multi-chain binding pockets is significantly lower than the variability of single-chain binding pockets, whereas there is no such pattern in heteromers (B). Monomers (C) show a pattern comparable to SBS homomers.

(D) The comparison of apo and holo structures of homomers indicates that the binding pockets MBS homomers do not show a larger structural variability than SBS homomers.

$^{**}p < 0.005$; $^{*}p < 0.05$; Wilcoxon tests with Benjamini-Hochberg correction.

accessible surface area (A_{rel}). Essentially, A_{rel} is the ratio of the solvent accessible surface area observed for a polypeptide chain (ignoring intermolecular interactions) to the value expected for a folded protein of the same molecular weight (Marsh and Teichmann, 2011). Previously, A_{rel} parameter has been shown to correlate very well with several more complex measures of protein flexibility as well as the magnitude of conformational changes that occur upon binding. Thus, it provides a simple way to analyze the flexibilities of protein complex subunits on a large scale. Interestingly, we observe that MBS complexes are generally more flexible than SBS complexes (Figure 6A). Previously, it was demonstrated that the subunits of protein complexes, heteromers in particular, are more flexible in their unbound states than monomers (Marsh and Teichmann, 2014) and that subunits of complexes with different symmetry types have different flexibilities (Marsh and Teichmann, 2014), i.e., cyclic homomers are more flexible than dihedral homomers. A comparison of symmetry types of homomers nevertheless indicates that the higher flexibility of MBS homomers is not a byproduct of biases in symmetry: MBS homomers are not consistently enriched among complexes with cyclic symmetry (Figure 6B). The biological function of the higher flexibility of MBS complexes is unclear; one possibility is that it is the consequence of the somewhat larger ligands of MBS complexes, which may require larger conformational changes upon binding.

Prokaryotes and eukaryotes are characterized with different frequencies of homomers and heteromers, with homomers being much more frequent in prokaryotes (Lynch, 2012; Marsh et al., 2015; Figure 6C). We hypothesized that, due to much stronger selection in prokaryotes, complexes where the topology is more influenced by stochastic processes, i.e., SBS complexes, will have a lower frequency in prokaryotes. Our results show that the frequency of SBS complexes is indeed significantly (although not dramatically) lower in prokaryotes than in eukaryotes both in the case of homomers and heteromers (Figure 5D), supporting the hypothesis that SBS complexes are subject to weaker selective constraints.

Pathogenic Mutations Are Most Enriched in the Binding Sites of Heteromers

Finally, we tested whether quaternary structure and the structure of binding pockets influences the pathogenicity of mutations in the binding sites. Proteins with different quaternary structure have different baseline levels of pathogenic mutations, which increase from monomers through homomers to heteromers, with MBS complexes having higher frequencies of pathogenic mutations than SBS complexes, both in homomers and heteromers (Figure 7; baseline is indicated with a red horizontal line). Mutations in binding sites are known to be more pathogenic than other mutations, and our findings confirm this; in most complex types, the frequency of pathogenic mutations is significantly higher than the baseline level ($^{*}p < 0.05$; $^{**}p < 0.005$; tests of proportions). The highest frequencies of pathogenic mutations are in the binding sites of heteromers (Figure 7B), which in the case of small-molecule binding MBS heteromers, is close to 30% of mutations. In the case of metal-binding MBS homomers, the total number of pathogenic mutations is very low, altogether 5, which results in low statistical power (Table S4).

DISCUSSION

Our findings indicate that the structure of ligand binding site, i.e., whether it is formed by residues of a single chain or by multiple chains, has profound consequences for the evolution of protein function and, in the case of cofactor or metal-binding proteins, also for quaternary structure. In the case of SBS complexes, the change in ligand binding follows qualitatively the same

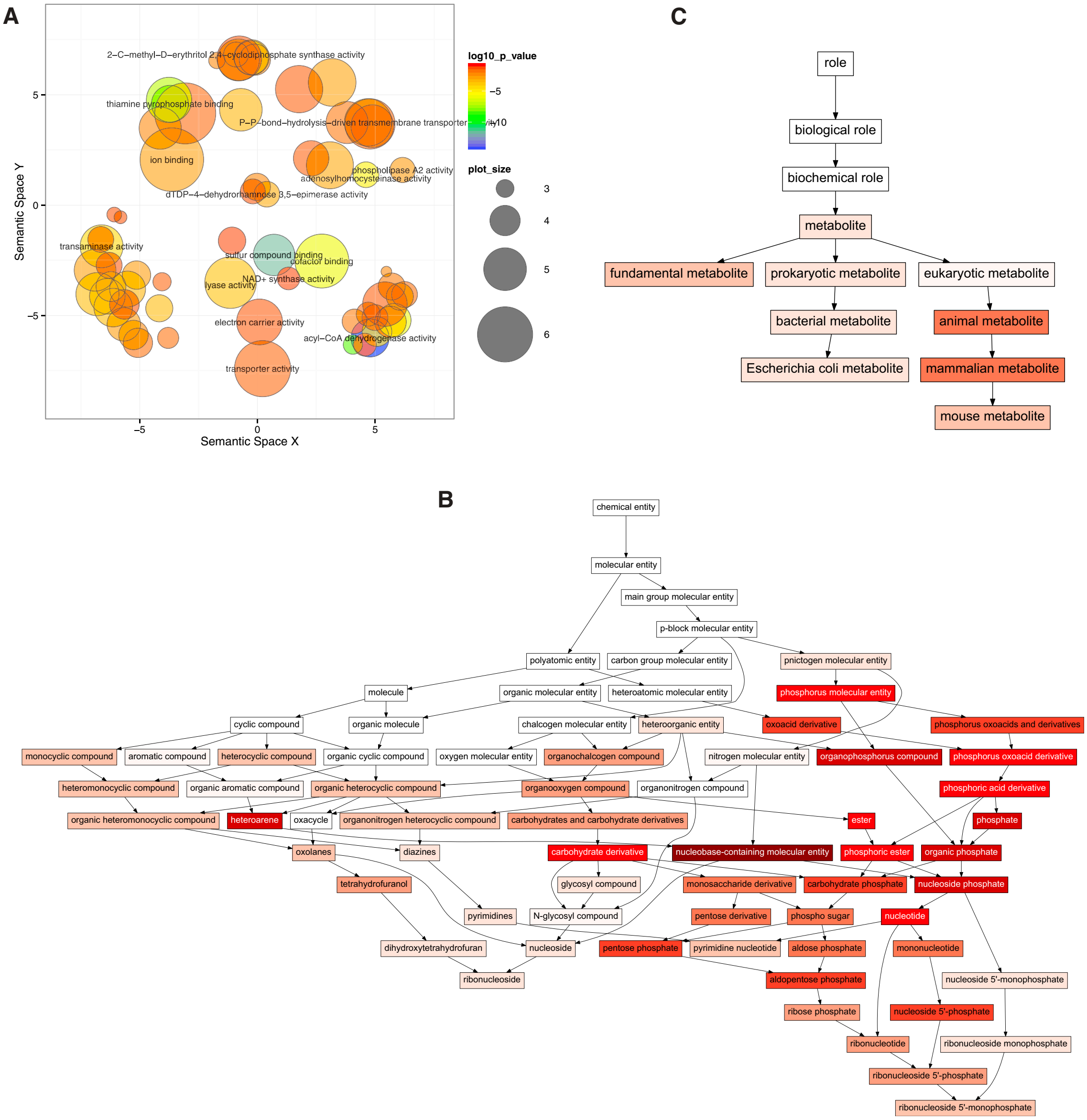

Figure 5. Functional Characteristics of MBS Homomers, in Relation to All Homomers included in the Analysis

(A) Scatterplot of significantly enriched GO molecular function terms, summarized and visualized with REVIGO. Related terms form clusters that are labeled with the most significant term of the cluster. The size of the circles corresponds to the number of proteins in the term; colors indicate significance. (See also Figure S5 and Table S1 for all enriched terms and exact significances.) MBS homomers have diverse functions, including acyl-CoA dehydrogenase activity, transaminase activity, thiamine pyrophosphate binding, cofactor binding, transmembrane transport, ion binding, or transporter activity, which frequently involve binding of cofactors.

(B) Graph of significantly enriched ChEBI structural ontology terms. White nodes are not significant; the intensity of red corresponds to significance (see Table S2 for exact p values). Most enriched structural terms are related to nucleobases/nucleotides.

(C) Graph of significantly enriched ChEBI role ontology terms. (See Table S3 for exact p values.) The enrichment shows that the ligands of MBS homomers are typically involved in metabolism.

pattern as in monomers, indicating that, in the case of such complexes, ligand binding is influenced little by quaternary structure and the evolution of quaternary structure is likely to be more influenced by stochastic processes, as it was suggested by Lynch (2013; Figure 2). Additionally, among all complex types, the baseline frequency of pathogenic mutations is the lowest in SBS complexes (Figure 7), further supporting this hypothesis. In contrast, in complexes that bind metals and cofactors, the quaternary structure of both MBS homomers and MBS heteromers evolves at a significantly different rate than in SBS complexes. Surprisingly, we found no evidence that changes in quaternary structure are a source of evolutionary innovations in

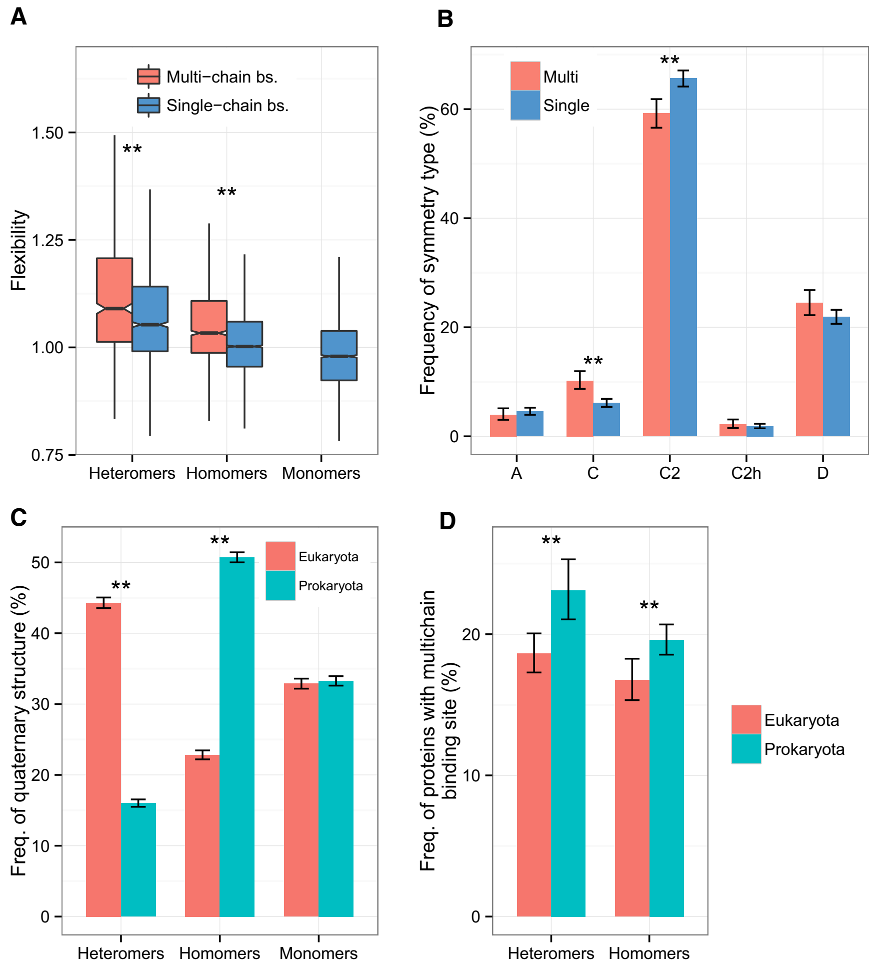

Figure 6. Structural and Evolutionary Characteristics of Single- and Multi-chain Binding Complexes

(A) Subunits forming MBSs are significantly more flexible than subunits with single-chain sites (Wilcoxon tests).

(B) The frequency of single- and multi-chain binding sites in different symmetry groups of homomers. The symmetry groups are A, asymmetric; C, cyclic; C2, two-fold dimeric; C2h, two-fold symmetric with >2 subunits; and D, dihedral (tests of proportions).

(C) The frequency of homomers is higher among prokaryotes, whereas heteromers are more frequent in eukaryotes (tests of proportions).

(D) The frequency of complexes with MBSs is higher in prokaryotes than in eukaryotes, particularly in the case of heteromers (tests of proportions). On (B)–(D), whiskers represent 95% confidence intervals. On all panels, $^{**}p < 0.005$ and $^{*}p < 0.05$.

homomers (at least when considering ligand binding): both ligand binding and the ligands of MBS homomers change significantly less with sequence evolution than in monomers (Figures 2A and 3A). The higher variability of quaternary structure (i.e., unit number) in cofactor-binding SBS homomers (Figure 2K) also suggests that purifying selection is more important in shaping the quaternary structure of MBS homomers than of SBS homomers.

In the case of cofactor-binding heteromers, we see the opposite pattern: the unit number of MBS complexes changes faster than the unit number of SBS complexes, indicating that, in such complexes, changes in complex topology are a source of evolutionary innovations (Figures 2L and S2). However, for heteromers that do not bind cofactors or metals (Figure S2), it is currently unclear to what degree the observed patterns are caused by real changes in function or the incompleteness of heteromeric structures in the PDB, and they probably require additional, more complete structures and more fine-grained analyses to be able to reach a conclusion.

The finding that the evolution and assembly of SBS complexes may be significantly influenced by non-adaptive processes does not mean that it is entirely stochastic—we observe a significantly higher baseline level of pathogenic mutation in SBS homomers than monomers (Figure 7), and the quaternary structure of such homomers is still frequently conserved (Levy et al., 2008; see also Figures 2 and S2). However, in addition to adaptive forces, the nonlinear change of tertiary structure with sequence similarity may contribute to the pattern. Typically, above ~40% sequence identity, the tertiary structure of homologous proteins differs little (see Abrusán and Marsh, 2016 for examples), and structural similarity declines significantly only below ~30% sequence identity. In fact, structure is so much more conserved than sequence that homology-based tools for tertiary structure prediction like Rosetta (Kim et al., 2004) or I-TASSER (Yang et al., 2015) can frequently build reliable predictions based on templates with as low as 15% sequence identity. Thus, similar quaternary structure of homologs might be, to some degree, the byproduct of their similar tertiary structure: similar building blocks result in similar complexes.

The observation that ligand structure and ligand pocket structure changes much less with sequence in MBS than SBS homomers is likely to have important consequences for drug design: the development of broad-spectrum antibiotics and antiviral drugs; for polypharmacology (Bolognesi and Cavalli, 2016); and also drug repositioning. Our findings suggest that antibiotics targeting MBS homomers are likely to be broader spectrum than antibiotics that target SBS homomers. In addition, it is likely that evolving resistance to drugs that target MBS homomers is more difficult, at least where the evolution of resistance is due to the accumulation of mutations, like in rapidly evolving retroviruses. The traditional aim of drug design has been designing drugs that bind specifically to one target protein (monopharmacology) to achieve maximum specificity and minimize side effects. However, it has become clear that many diseases, like cancers and psychiatric diseases, have polygenic causes, and in such

OPEN
ACCESS
CellPress

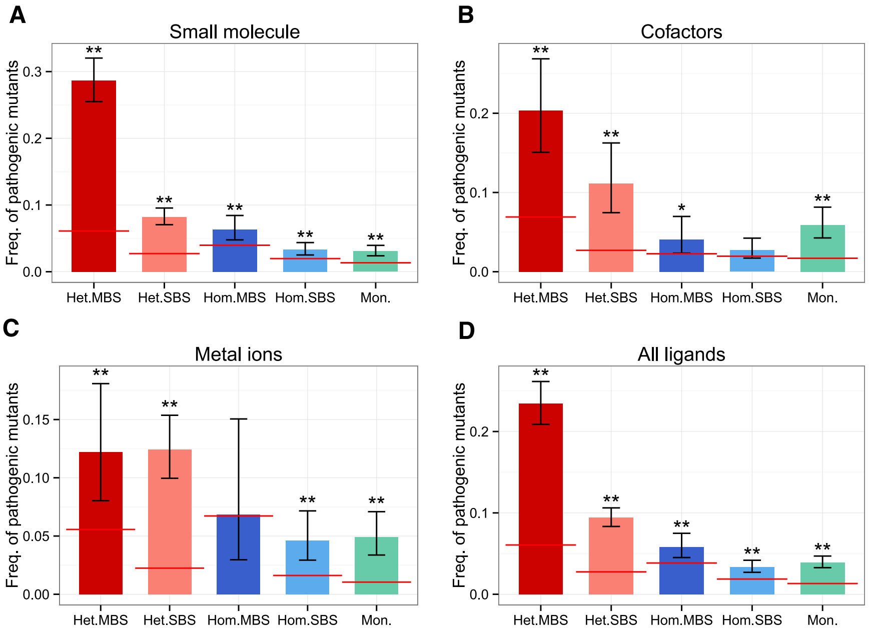

Figure 7. The Frequency of Pathogenic Mutations Is Different in Different Complexes

(A–D) Complexes with (A) small molecules, (B) cofactors, (C) metal ions, and (D) all ligands. The baseline level of pathogenic mutations is highest in MBS heteromers and lowest in monomers. In most complex types, the frequency of pathogenic mutations of the binding sites is higher than the baseline level, particularly in the case of heteromers. $^{**}p < 0.005$; $^{*}p < 0.05$; tests of proportions; whiskers represent 95% confidence intervals.

diseases, drugs that target several proteins simultaneously (multi-target or promiscuous drugs) can be much more efficient than single-target drugs, despite their more complex and less understood pharmacology (Peters, 2013; Roth et al., 2004). Because similar binding pockets in general bind similar ligands (Klabunde, 2007), protein families with many MBS homomers are likely to be good candidates for the development of multi-target drugs, particularly in cancers, due to the specific metabolic characteristics of many cancers (Cairns et al., 2011). Finally, our results may be also relevant for drug repositioning (Li and Jones, 2012), because binding site similarity is a requirement for repositioned drugs. An example is the retroviral drug nelfinavir, which binds HIV protease, a homomer with a MBS, and is currently being repositioned as a (promiscuous) cancer drug (Koltai, 2015).

EXPERIMENTAL PROCEDURES

Data Sources and Data Preprocessing

The sequences of proteins having a structure in the PDB were downloaded from Uniprot, and we used the Uniprot mappings between sequences and PDB structures. To identify homologous protein pairs, we performed an all versus all BLAST search on these sequences, with an e-value cutoff 10^{-4}; the sequence pairs with significant BLAST hits were realigned with Muscle (Edgar, 2004), and their global sequence similarity was determined. Chimeric sequences were excluded from further analyses, and the sequences were clustered at 100% sequence similarity to remove redundancies. For every protein sequence, we determined the PDB entries associated with it and filtered out the PDB entries that are part of a virus, form protein fibrils, are helical, or contain sequences from more than one species (this also removes most peptide-binding antibodies). The quaternary structure of the proteins was determined using the biological units as follows: if a protein is part of at least one heteromer PDB structure, it was classified as a heteromer; else, if it has at least one homomeric PDB entry, it was classified as a homomer, and the remaining sequences were classified as monomers. Heteromer PDB structures were defined as structures with chains from minimum two different proteins, irrespectively of the length of the chains.

We used the BioLiP database (Yang et al., 2013) of protein-ligand interactions to determine ligand binding residues and whether a ligand is bound to several protein chains or a single one. BioLiP is a semi-manually curated database, which contains only ligands that are assumed to be biologically relevant and are not byproducts of the crystallization procedure or other structure-determination methods. It contains all structures from the PDB with a biologically relevant ligand (at the time of writing 71,925 structures, after excluding structures binding only nucleic acids and peptides), but it is based on the asymmetric units. Because in the analyses, we used the biological units of PDB entries (the functional form of the structures), we excluded all entries, altogether 13,413, where the asymmetric unit and biounit is different and the number of proteins is larger in the biounit than in the asymmetric unit, as the ligand annotation of these entries is incomplete in BioLiP. In the case of entries where the asymmetric units contain the biounit, differences between the two are incorporated into the BioLiP annotation; see Yang et al. (2013) for details.

From the ligands of BioLiP, we used cofactors, metal ions, and small molecules. Cofactors were determined as ligands that are annotated as cofactors in the ChEBI database (Hastings et al., 2016) or in Fischer et al. (2010). Small molecules were defined as every ligand that is not a cofactor, metal ion, nucleic acid, or a peptide.

Searches for Ligand Binding Sites

We used ProBiS (Konc and Janežič, 2010, 2017) to identify ligand binding pockets in homologous proteins, which identifies binding sites by local structural search and compositional similarity. We performed all versus all searches between homologous protein pairs; i.e., we searched all structures of the target protein with all ligands of all structures of the query protein (Figure 1E). In the first step, the biological units of every PDB entry were preprocessed: their atoms and chains were renumbered so that every atom and protein chain was unique in the structure, and from the target protein structures, all ligands were removed, whereas from the query structures, only waters. Due to the limitations of PDB format, structures with more than 99,999 atoms were not included in the analyses. Next, the binding sites of ligands present in BioLiP were extracted from the query structures, which were defined as the surface residues within 3 Å of the ligand. For the target structures, the entire surface of the protein (complex) was extracted, and the binding sites were searched against the surfaces of the target proteins with ProBiS. For every hit, the Z score, RMSD, e-value, and the size of the match were determined (size was determined as the number of aligned vertices; see Konc and Janežič, 2010 for details). We accepted a hit as significant if the Z score of the match was equal or higher than 2 and its e-value was lower than 10^{-4}. The fraction of different binding sites for a pair of homologs was determined as the number of ligands without a significant hit in the homolog divided by the total number of ligands searched and was plotted against the sequence similarity of the homologous proteins. In addition, we tested how efficient is

ProBiS in finding matches and whether there are systematic differences between homomers, heteromers, and monomers by determining the fraction of significant hits between homologous structures that are known to bind the same ligand (Figure S7). We found that the efficiency of ProBiS is high and is not influenced qualitatively by the size or number of chains in a PDB entry (Figure S7).

Determination of Ligand Chemical Similarity and Binding Site Variability

We used the obo ontology files downloaded from the ChEBI database (Degtyarenko et al., 2008; Hastings et al., 2016) to compare the chemical similarities of ligands. For every ligand, its entire structural ontology graph was computed from the obo files using a recursive algorithm, using the "is_a" tag, and the average of the shared/different terms was determined for every possible ligand pair of the homologous sequences.

Structural variability of the binding sites of homologs was performed using the RMSD of the significant hits (Z score $\geq$ 2), using the sequence pairs with 50%–30% identity. We used the size of the match as a covariate because RMSD is not a size-independent measure; it was measured as the number of aligned ProBiS vertices. We performed similar searches between the ligand binding (holo) structures and ligand-free (apo) structures of the same proteins when they were both available in the PDB. In these searches, no significance cutoff was used. Variability in the structure of the binding sites was measured as the average RMSD of the best hits, using the size of the match as covariate.

Gene Ontology and ChEBI Ontology Enrichment

From the UniProt-CrossRef annotation of proteins that are present in the PDB, we extracted the list of GO terms associated with a particular protein. Next, for every protein, we extracted the entire hierarchy of its GO terms, i.e., all parents up to the highest level "molecular function" term, and determined the enrichment of GO terms in MBS homomers with GeneMerge (Castillo-Davis and Hartl, 2003), using all homomers in the PDB as the background set. Significances were corrected for multiple testing with the Benjamini-Hochberg method (a.k.a. false discovery rate [FDR]), and the list of significantly enriched terms was submitted to the Revigo server (Supek et al., 2011) to remove redundancies, summarize, and visualize the results.

ChEBI term enrichment was determined as follows. We determined the nonredundant list of ligands in MBS homomers and in all homomers, containing only a single instance of every ligand. For every ligand, we identified its parental terms using the ChEBI structural ("is_a") and role ("has_role") ontologies. The enrichment of terms was also calculated with GeneMerge, which is a generic tool for term-enrichment analysis of ranked lists. Enriched terms (after correction for multiple testing with FDR) were visualized with a method conceptually similar to the graphical output of GOrilla (Eden et al., 2009), with directed graphs and using color coding to indicate the significance of the enrichment, the intensity of red indicating significance. Graphs were drawn using GraphViz.

Evolutionary and Structural Characteristics of Complexes

The evolutionary age of human proteins was based on the ProteinHistorian database (Capra et al., 2012), using the Princeton Protein Orthology (PPOD)/PANTHERv7 dataset, generated with asymmetric Wagner parsimony. The flexibility of subunits of proteins was determined as described in Marsh and Teichmann (2014); the symmetry type of homomeric complexes is based on the classification of PDB.

Estimation of the Frequency of Pathogenic Mutants

The list of disease-causing mutations was downloaded from Ensembl (variation dataset) and was mapped to the PDB structures of human proteins or their homologs if the sequence similarity between the human protein and its homolog was higher than 90% and the mutated amino acid was identical. The set of putative neutral mutations is based on ExAC variants (Lek et al., 2016) that map to structures of the PDB using a similar procedure as described above. The frequency of pathogenic mutants—both baseline and binding—was determined for every protein type independently as the number of disease mutations divided by the sum of disease and neutral mutations.

Analysis Tools and Statistics

All analyses and statistical tests (except GO and ChEBI enrichment) were performed with in-house Perl scripts and R. Protein structures were visualized with UCSF Chimera (Pettersen et al., 2004).

DATA AND SOFTWARE AVAILABILITY

Supplemental data are available from Edinburgh Datashare (https://datashare.is.ed.ac.uk/handle/10283/3024).

SUPPLEMENTAL INFORMATION

Supplemental Information includes seven figures and four tables and can be found with this article online at https://doi.org/10.1016/j.celrep.2018.02.085.

ACKNOWLEDGMENTS

We thank Janez Konc for help and suggestions on ProBiS usage and Jonathan Wells and the anonymous reviewers for helpful comments on the manuscript. We acknowledge the use of the Eddie3 computing cluster of the University of Edinburgh and thank for the support from the Research Computing staff. This work was supported by the Medical Research Council (Career Development Award MR/M02122X/1 to J.A.M.).

AUTHOR CONTRIBUTIONS

G.A. conceived the project, performed the analyses, and wrote the first version of the manuscript. J.A.M. contributed to the analysis and the writing of the manuscript.

DECLARATION OF INTERESTS

The authors declare no competing interests.

Received: July 5, 2017
Revised: January 17, 2018
Accepted: February 22, 2018
Published: March 20, 2018

REFERENCES

Abrusán, G., and Marsh, J.A. (2016). Alpha helices are more robust to mutations than beta strands. PLoS Comput. Biol. *12*, e1005242.

Ahnert, S.E., Marsh, J.A., Hernández, H., Robinson, C.V., and Teichmann, S.A. (2015). Principles of assembly reveal a periodic table of protein complexes. Science *350*, aaa2245.

Ali, M.H., and Imperiali, B. (2005). Protein oligomerization: how and why. Bioorg. Med. Chem. *13*, 5013–5020.

Bergendahl, L.T., and Marsh, J.A. (2017). Functional determinants of protein assembly into homomeric complexes. Sci. Rep. *7*, 4932.

Bolognesi, M.L., and Cavalli, A. (2016). Multitarget drug discovery and polypharmacology. ChemMedChem *11*, 1190–1192.

Cairns, R.A., Harris, I.S., and Mak, T.W. (2011). Regulation of cancer cell metabolism. Nat. Rev. Cancer *11*, 85–95.

Capra, J.A., Williams, A.G., and Pollard, K.S. (2012). ProteinHistorian: tools for the comparative analysis of eukaryote protein origin. PLoS Comput. Biol. *8*, e1002567.

Capra, J.A., Stolzer, M., Durand, D., and Pollard, K.S. (2013). How old is my gene? Trends Genet. *29*, 659–668.

Castillo-Davis, C.I., and Hartl, D.L. (2003). GeneMerge–post-genomic analysis, data mining, and hypothesis testing. Bioinformatics *19*, 891–892.

Degtyarenko, K., de Matos, P., Ennis, M., Hastings, J., Zbinden, M., McNaught, A., Alcántara, R., Darsow, M., Guedj, M., and Ashburner, M. (2008). ChEBI: a

database and ontology for chemical entities of biological interest. Nucleic Acids Res. *36*, D344–D350.

Eden, E., Navon, R., Steinfeld, I., Lipson, D., and Yakhini, Z. (2009). GOrilla: a tool for discovery and visualization of enriched GO terms in ranked gene lists. BMC Bioinformatics *10*, 48.

Edgar, R.C. (2004). MUSCLE: multiple sequence alignment with high accuracy and high throughput. Nucleic Acids Res. *32*, 1792–1797.

Fischer, J.D., Holliday, G.L., Rahman, S.A., and Thornton, J.M. (2010). The structures and physicochemical properties of organic cofactors in biocatalysis. J. Mol. Biol. *403*, 803–824.

Fong, D.H., Burk, D.L., Blanchet, J., Yan, A.Y., and Berghuis, A.M. (2017). Structural basis for kinase-mediated macrolide antibiotic resistance. Structure *25*, 750–761.e5.

Furnham, N., Dawson, N.L., Rahman, S.A., Thornton, J.M., and Orengo, C.A. (2016). Large-scale analysis exploring evolution of catalytic machineries and mechanisms in enzyme superfamilies. J. Mol. Biol. *428* (*2 Pt A*), 253–267.

Hastings, J., Owen, G., Dekker, A., Ennis, M., Kale, N., Muthukrishnan, V., Turner, S., Swainston, N., Mendes, P., and Steinbeck, C. (2016). ChEBI in 2016: improved services and an expanding collection of metabolites. Nucleic Acids Res. *44* (*D1*), D1214–D1219.

Hvorecny, K.L., Bahl, C.D., Kitamura, S., Lee, K.S.S., Hammock, B.D., Morisseau, C., and Madden, D.R. (2017). Active-site flexibility and substrate specificity in a bacterial virulence factor: crystallographic snapshots of an epoxide hydrolase. Structure *25*, 697–707.e4.

Ji, H.-F., Kong, D.-X., Shen, L., Chen, L.-L., Ma, B.-G., and Zhang, H.-Y. (2007). Distribution patterns of small-molecule ligands in the protein universe and implications for origin of life and drug discovery. Genome Biol. *8*, R176.

Johnson, Z.L., and Chen, J. (2017). Structural basis of substrate recognition by the multidrug resistance protein MRP1. Cell *168*, 1075–1085.e9.

Kim, D.E., Chivian, D., and Baker, D. (2004). Protein structure prediction and analysis using the Robetta server. Nucleic Acids Res. *32*, W526–W531.

Kinjo, A.R., and Nakamura, H. (2009). Comprehensive structural classification of ligand-binding motifs in proteins. Structure *17*, 234–246.

Klabunde, T. (2007). Chemogenomic approaches to drug discovery: similar receptors bind similar ligands. Br. J. Pharmacol. *152*, 5–7.

Koltai, T. (2015). Nelfinavir and other protease inhibitors in cancer: mechanisms involved in anticancer activity. F1000Res. *4*, 9.

Konc, J., and Janežič, D. (2010). ProBiS algorithm for detection of structurally similar protein binding sites by local structural alignment. Bioinformatics *26*, 1160–1168.

Konc, J., and Janežič, D. (2017). ProBiS tools (algorithm, database, and web servers) for predicting and modeling of biologically interesting proteins. Prog. Biophys. Mol. Biol. *128*, 24–32.

Lek, M., Karczewski, K.J., Minikel, E.V., Samocha, K.E., Banks, E., Fennell, T., O'Donnell-Luria, A.H., Ware, J.S., Hill, A.J., Cummings, B.B., et al.; Exome Aggregation Consortium (2016). Analysis of protein-coding genetic variation in 60,706 humans. Nature *536*, 285–291.

Levy, E.D., and Teichmann, S. (2013). Structural, evolutionary, and assembly principles of protein oligomerization. Prog. Mol. Biol. Transl. Sci. *117*, 25–51.

Levy, E.D., Boeri Erba, E., Robinson, C.V., and Teichmann, S.A. (2008). Assembly reflects evolution of protein complexes. Nature *453*, 1262–1265.

Li, Y.Y., and Jones, S.J. (2012). Drug repositioning for personalized medicine. Genome Med. *4*, 27.

Lynch, M. (2007). The Origins of Genome Architecture (Sunderland, Mass: Sinauer Associates).

Lynch, M. (2012). The evolution of multimeric protein assemblages. Mol. Biol. Evol. *29*, 1353–1366.

Lynch, M. (2013). Evolutionary diversification of the multimeric states of proteins. Proc. Natl. Acad. Sci. USA *110*, E2821–E2828.

Lynch, M., and Conery, J.S. (2003). The origins of genome complexity. Science *302*, 1401–1404.

Marianayagam, N.J., Sunde, M., and Matthews, J.M. (2004). The power of two: protein dimerization in biology. Trends Biochem. Sci. *29*, 618–625.

Marsh, J.A., and Teichmann, S.A. (2011). Relative solvent accessible surface area predicts protein conformational changes upon binding. Structure *19*, 859–867.

Marsh, J.A., and Teichmann, S.A. (2014). Protein flexibility facilitates quaternary structure assembly and evolution. PLoS Biol. *12*, e1001870.

Marsh, J.A., and Teichmann, S.A. (2015). Structure, dynamics, assembly, and evolution of protein complexes. Annu. Rev. Biochem. *84*, 551–575.

Marsh, J.A., Hernández, H., Hall, Z., Ahnert, S.E., Perica, T., Robinson, C.V., and Teichmann, S.A. (2013). Protein complexes are under evolutionary selection to assemble via ordered pathways. Cell *153*, 461–470.

Marsh, J.A., Rees, H.A., Ahnert, S.E., and Teichmann, S.A. (2015). Structural and evolutionary versatility in protein complexes with uneven stoichiometry. Nat. Commun. *6*, 6394.

Nath, N., Mitchell, J.B.O., and Caetano-Anollés, G. (2014). The natural history of biocatalytic mechanisms. PLoS Comput. Biol. *10*, e1003642.

Nishi, H., Hashimoto, K., Madej, T., and Panchenko, A.R. (2013). Evolutionary, physicochemical, and functional mechanisms of protein homooligomerization. Prog. Mol. Biol. Transl. Sci. *117*, 3–24.

Osadchy, M., and Kolodny, R. (2011). Maps of protein structure space reveal a fundamental relationship between protein structure and function. Proc. Natl. Acad. Sci. USA *108*, 12301–12306.

Pabon, N.A., and Camacho, C.J. (2017). Probing protein flexibility reveals a mechanism for selective promiscuity. eLife *6*, e22889.

Peters, J.-U. (2013). Polypharmacology - foe or friend? J. Med. Chem. *56*, 8955–8971.

Petsko, G., and Ringe, D. (2008). Protein Structure and Function (Oxford, New York: Oxford University Press).

Pettersen, E.F., Goddard, T.D., Huang, C.C., Couch, G.S., Greenblatt, D.M., Meng, E.C., and Ferrin, T.E. (2004). UCSF Chimera–a visualization system for exploratory research and analysis. J. Comput. Chem. *25*, 1605–1612.

Roth, B.L., Sheffler, D.J., and Kroeze, W.K. (2004). Magic shotguns versus magic bullets: selectively non-selective drugs for mood disorders and schizophrenia. Nat. Rev. Drug Discov. *3*, 353–359.

Sahni, N., Yi, S., Taipale, M., Fuxman Bass, J.I., Coulombe-Huntington, J., Yang, F., Peng, J., Weile, J., Karras, G.I., Wang, Y., et al. (2015). Widespread macromolecular interaction perturbations in human genetic disorders. Cell *161*, 647–660.

Smith, E., and Morowitz, H.J. (2016). The Origin and Nature of Life on Earth: The Emergence of the Fourth Geosphere (Cambridge University Press).

Stockwell, G.R., and Thornton, J.M. (2006). Conformational diversity of ligands bound to proteins. J. Mol. Biol. *356*, 928–944.

Supek, F., Bošnjak, M., Škunca, N., and Šmuc, T. (2011). REVIGO summarizes and visualizes long lists of gene ontology terms. PLoS ONE *6*, e21800.

Wells, J.N., Bergendahl, L.T., and Marsh, J.A. (2016). Operon gene order is optimized for ordered protein complex assembly. Cell Rep. *14*, 679–685.

White, H.B., 3rd. (1976). Coenzymes as fossils of an earlier metabolic state. J. Mol. Evol. *7*, 101–104.

Yang, J., Roy, A., and Zhang, Y. (2013). BioLiP: a semi-manually curated database for biologically relevant ligand-protein interactions. Nucleic Acids Res. *41*, D1096–D1103.

Yang, J., Yan, R., Roy, A., Xu, D., Poisson, J., and Zhang, Y. (2015). The I-TASSER Suite: protein structure and function prediction. Nat. Methods *12*, 7–8.

Yates, C.M., and Sternberg, M.J.E. (2013). The effects of non-synonymous single nucleotide polymorphisms (nsSNPs) on protein-protein interactions. J. Mol. Biol. *425*, 3949–3963.

Cell Reports, Volume *22*

Supplemental Information

Ligand Binding Site Structure Influences the Evolution of Protein Complex Function and Topology

György Abrusán and Joseph A. Marsh

SUPPLEMENTARY FIGURES

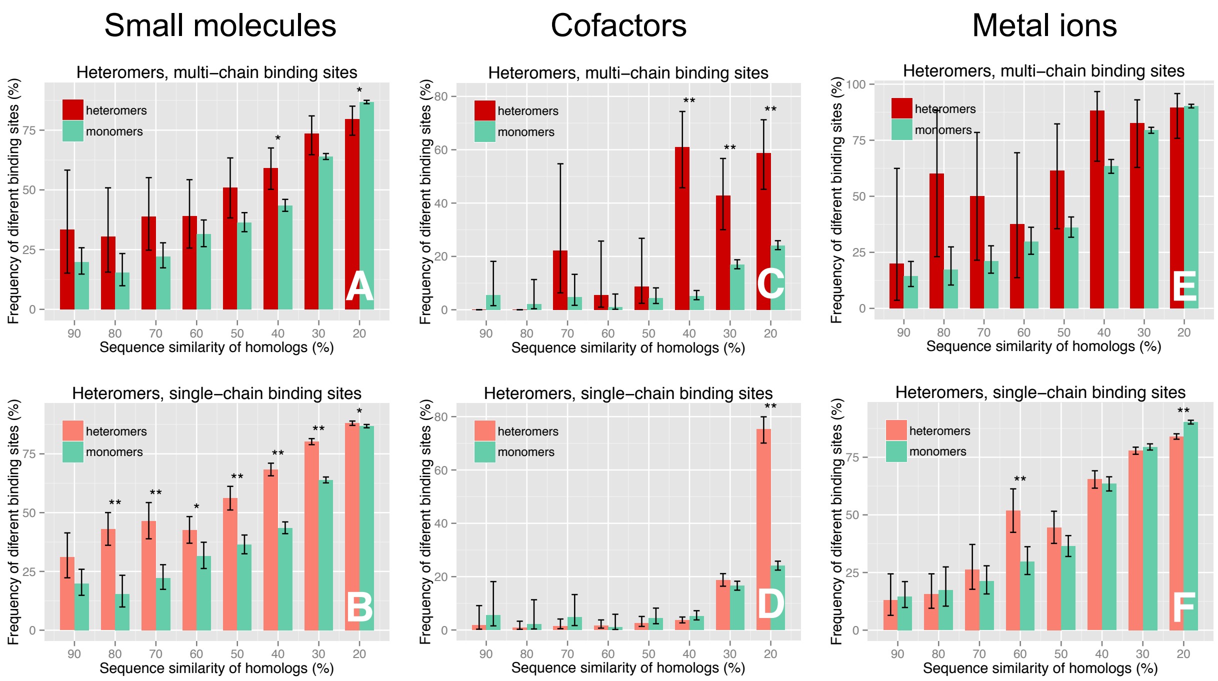

Figure S1, Related to Figure2. Evolution of ligand binding of small organic ligands (first column, panels **A**,**B**), cofactors (second column, panels **C**,**D**) and metal ions (third column, panels **E**,**F**) of heteromers. Whiskers represent 95% confidence intervals, whiskers for bins with zero frequency were not drawn; **: $p < 0.005$, *: $p < 0.05$; tests of proportions, with Benjamini-Hochberg correction for multiple testing.

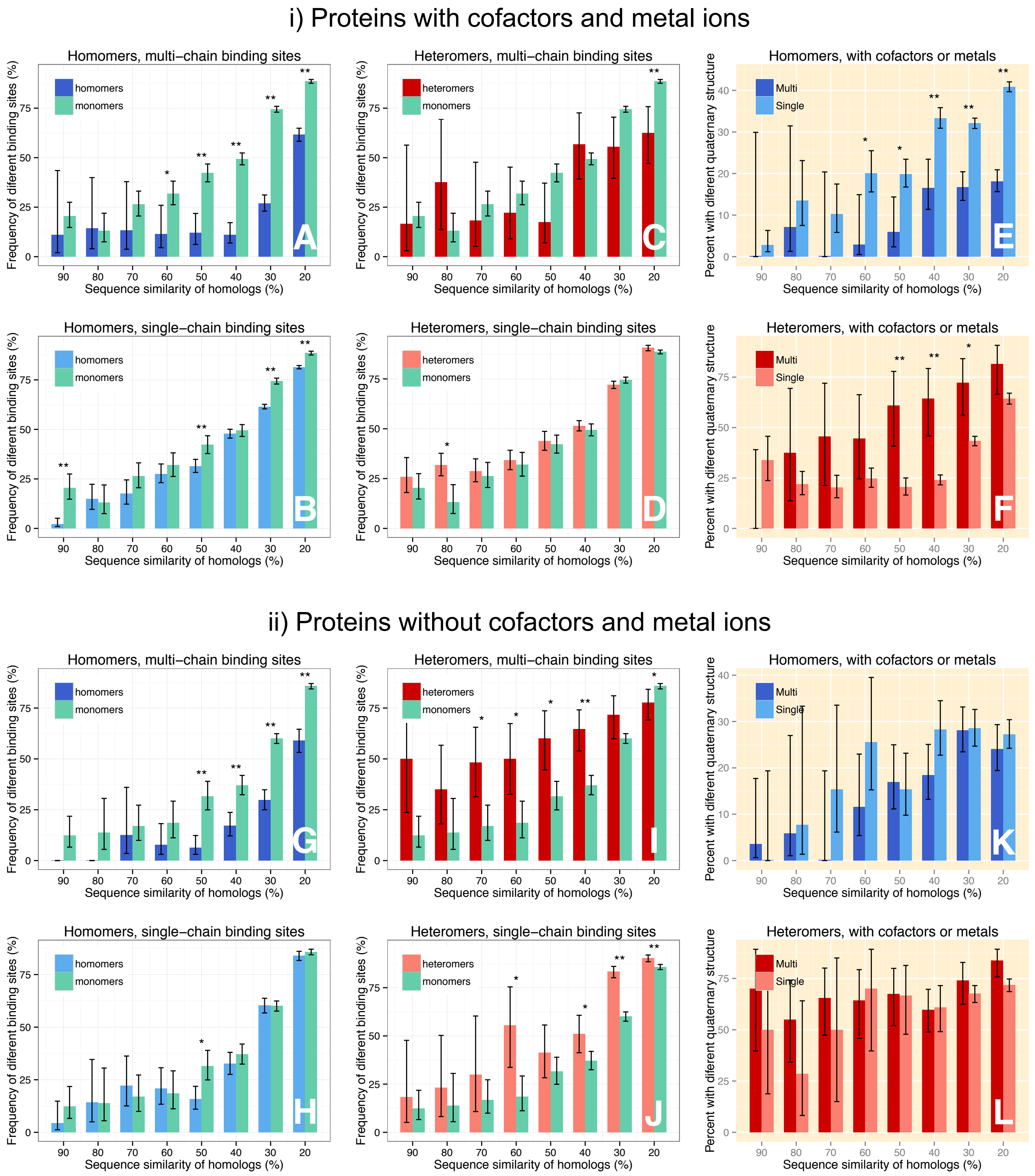

Figure S2, Related to Figure 2. Evolution of ligand binding and quaternary structure in proteins that bind metals or cofactors (panels **A-F**) and in proteins that do not (panels **G-L**). In the case of proteins with cofactors the conservation quaternary structure (unit number) of MBS and SBS complexes is markedly different for both homomers and heteromers (**E,F**), but show opposite patterns, while in the case of proteins without cofactors no significant difference exists (**K,L**). In the case of heteromers without cofactors (**L**) the lack of a positive correlation between sequence divergence and quaternary structure change suggests that the pattern is largely influenced by the incompleteness of structures in PDB. (Whiskers represent 95% confidence intervals, whiskers for bins with zero frequency wee not drawn; **: $p < 0.005$, *: $p < 0.05$; tests of proportions, with Benjamini-Hochberg correction for multiple testing.)

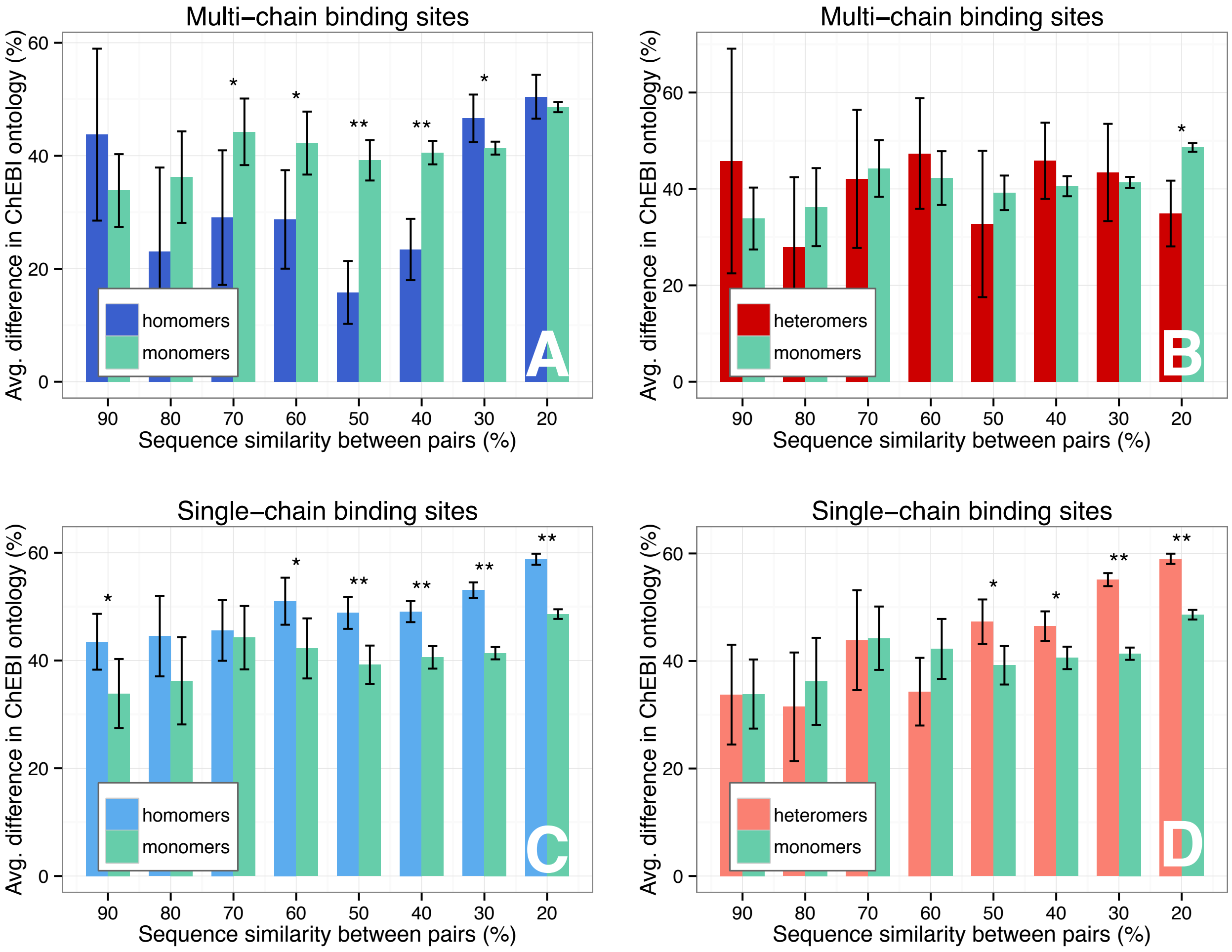

Figure S3. The change in chemical similarity of ligands, using only small molecules. Related to Figure 3. The pattern is qualitatively similar to the pattern shown on Figure 3, which includes also cofactors.

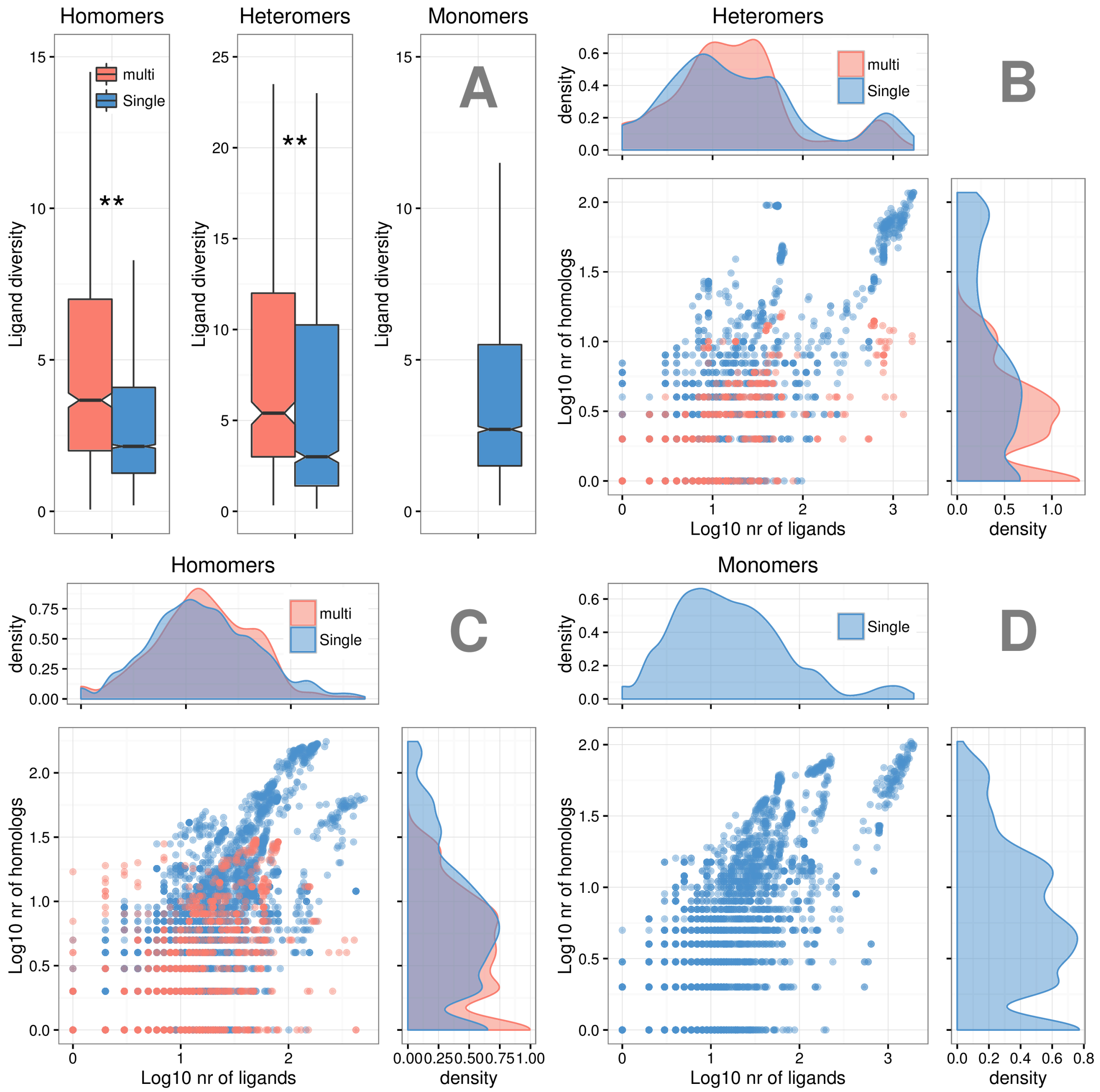

Figure S4. Complexes with multi-chain binding sites have more diverse ligand sets. Related to Figures 3 and 4. **A)** Ligand diversity (number of different ligands / number of homologous sequences) of different complex types. Note that the magnitude of the structural difference between different ligands was ignored, thus the differences quantify research effort, and not structural variability of ligands (for example, ATP and ADP are different ligands, although they are structurally very similar). Proteins with multi-chain binding sites have more ligands per sequence, and the difference is most pronounced in the case of homomers (**: $p < 0.005$, Wilcoxon tests). This indicates that the observed lower structural variability of ligands of homomers with multichain binding sites is not the consequence of systematic biases in ligand numbers, or the number of homologs in the PDB. Note that it is difficult to compare diversity between homomers and heteromers, because the latter have a much more variable sequence composition, resulting in higher overall ligand diversity. **B-D)** Variability in the number of homologous sequences and the number of different ligands of homomers, heteromers and monomers.

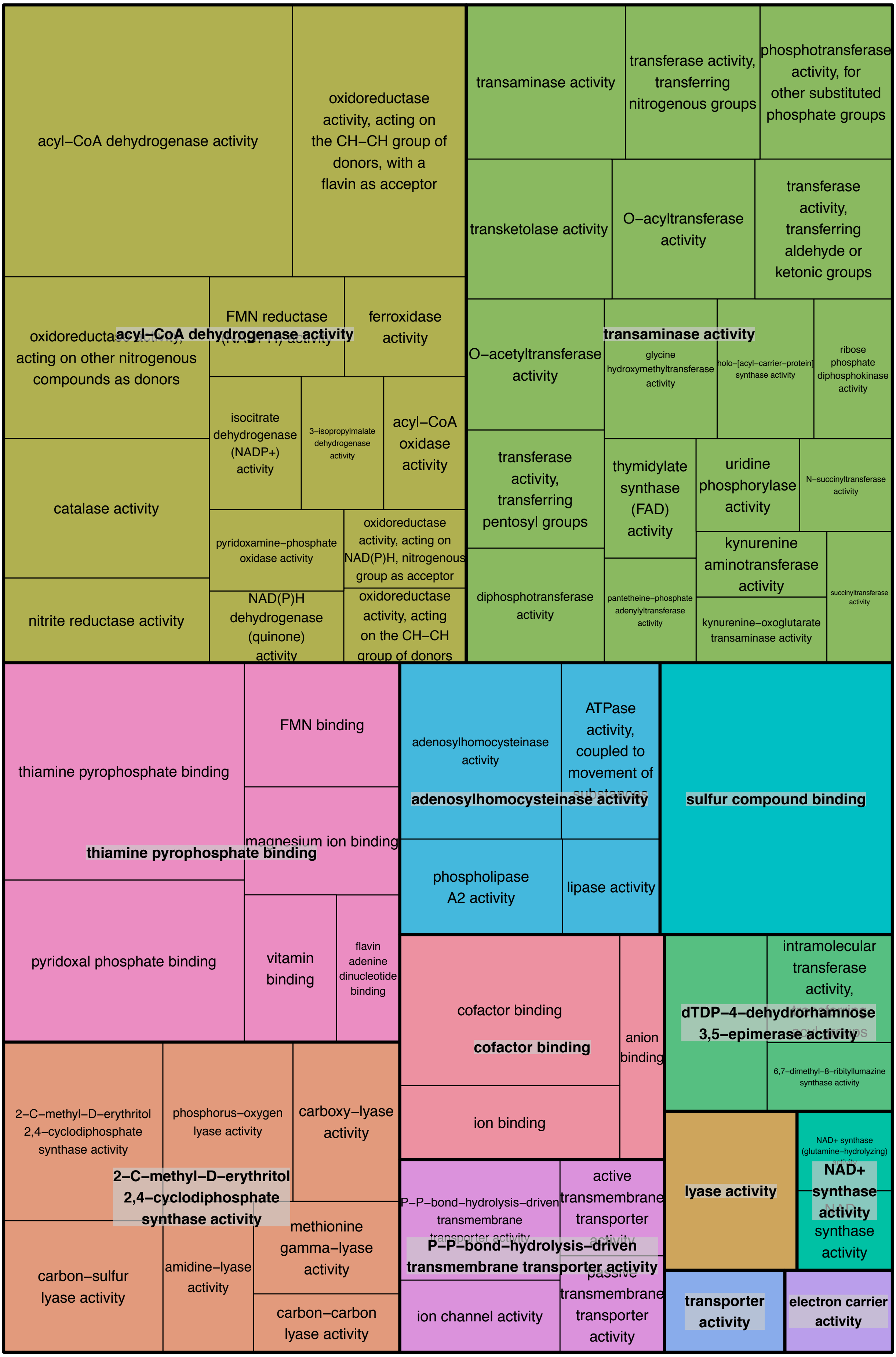
acyl−CoA dehydrogenase activity
oxidoreductase activity, acting on the CH−CH group of donors, with a flavin as acceptor
oxidoreductase activity, acting on other nitrogenous compounds as donors
acyl−CoA dehydrogenase activity
FMN reductase
ferroxidase activity
isocitrate dehydrogenase (NADP+) activity
3−isopropylmalate dehydrogenase activity
acyl−CoA oxidase activity
catalase activity
pyridoxamine−phosphate oxidase activity
oxidoreductase activity, acting on NAD(P)H, nitrogenous group as acceptor
nitrite reductase activity
NAD(P)H dehydrogenase (quinone) activity
oxidoreductase activity, acting on the CH−CH group of donors
transaminase activity
transferase activity, transferring nitrogenous groups
phosphotransferase activity, for other substituted phosphate groups
transketolase activity
O−acyltransferase activity
transferase activity, transferring aldehyde or ketonic groups
O−acetyltransferase activity
transaminase activity
glycine hydroxymethyltransferase activity
holo−[acyl−carrier−protein] synthase activity
ribose phosphate diphosphokinase activity
transferase activity, transferring pentosyl groups
thymidylate synthase (FAD) activity
uridine phosphorylase activity
N−succinyltransferase activity
kynurenine aminotransferase activity
diphosphotransferase activity
pantetheine−phosphate adenylyltransferase activity
kynurenine−oxoglutarate transaminase activity
succinyltransferase activity
thiamine pyrophosphate binding
FMN binding
magnesium ion binding
thiamine pyrophosphate binding
pyridoxal phosphate binding
vitamin binding
flavin adenine dinucleotide binding
adenosylhomocysteinase activity
ATPase activity, coupled to movement of substances
adenosylhomocysteinase activity
phospholipase A2 activity
lipase activity
sulfur compound binding
cofactor binding
cofactor binding
anion binding
ion binding
intramolecular transferase activity, transferring acyl groups
dTDP−4−dehydrorhamnose 3,5−epimerase activity
6,7−dimethyl−8−ribityllumazine synthase activity
2−C−methyl−D−erythritol 2,4−cyclodiphosphate synthase activity
phosphorus−oxygen lyase activity
carboxy−lyase activity
2−C−methyl−D−erythritol 2,4−cyclodiphosphate synthase activity
methionine gamma−lyase activity
carbon−sulfur lyase activity
amidine−lyase activity
carbon−carbon lyase activity
P−P−bond−hydrolysis−driven transmembrane transporter activity
active transmembrane transporter activity
P−P−bond−hydrolysis−driven transmembrane transporter activity
passive transmembrane transporter activity
ion channel activity
lyase activity
NAD+ synthase (glutamine−hydrolyzing) activity
NAD+ synthase activity
NAD+ synthase activity
transporter activity
electron carrier activity

Figure S5 (previous page). TreeMap of significantly overrepresented GO terms (molecular function) of MBS homomers. Related to Figure 5. The size of rectangles is proportional to the p-value of the terms, colors indicate clusters of related terms. See also Table S1.

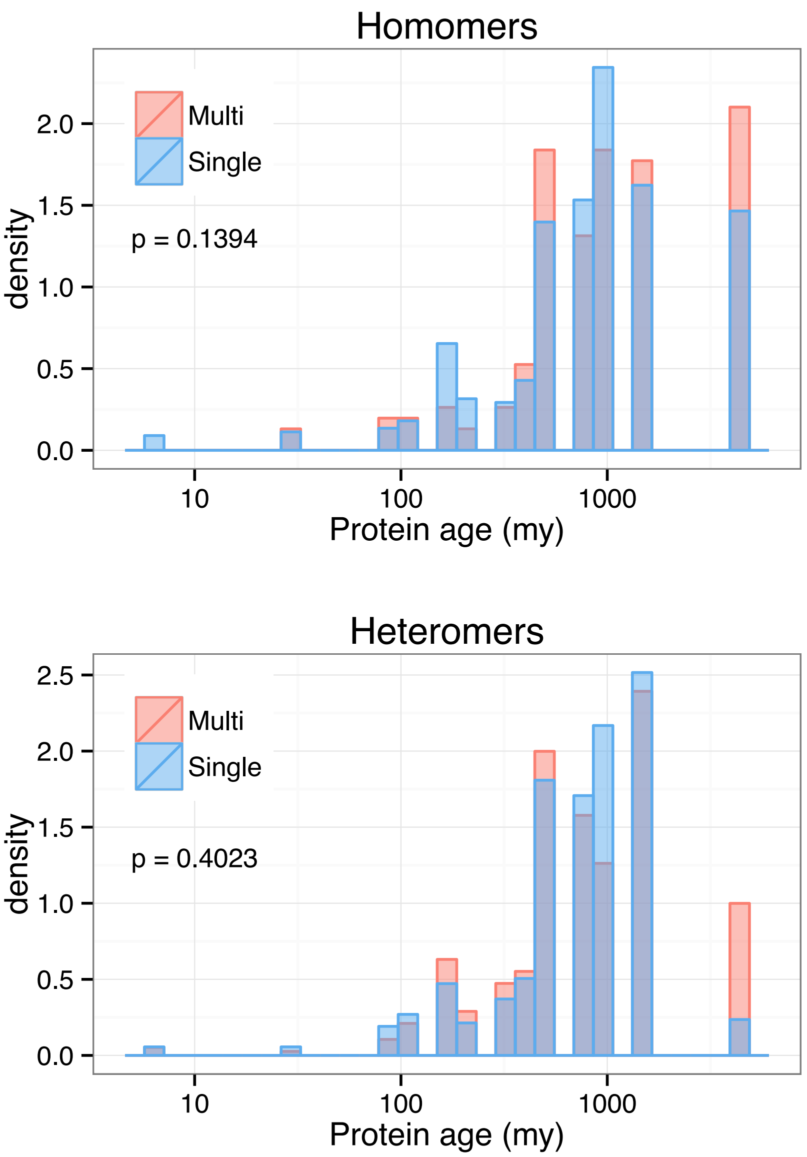

Figure S6, related to Figure 6. The age of proteins of complexes with MBS binding sites is not significantly different from the proteins of SBS complexes, although the difference between MBS and SBS proteins is largest in the oldest bin, representing proteins that were already present in the last universal common ancestor (LUCA) of all cellular organisms. Note that several complexes contain both types of binding sites, these are present in both distributions. Significance was tested with two sample Wilcoxon tests.

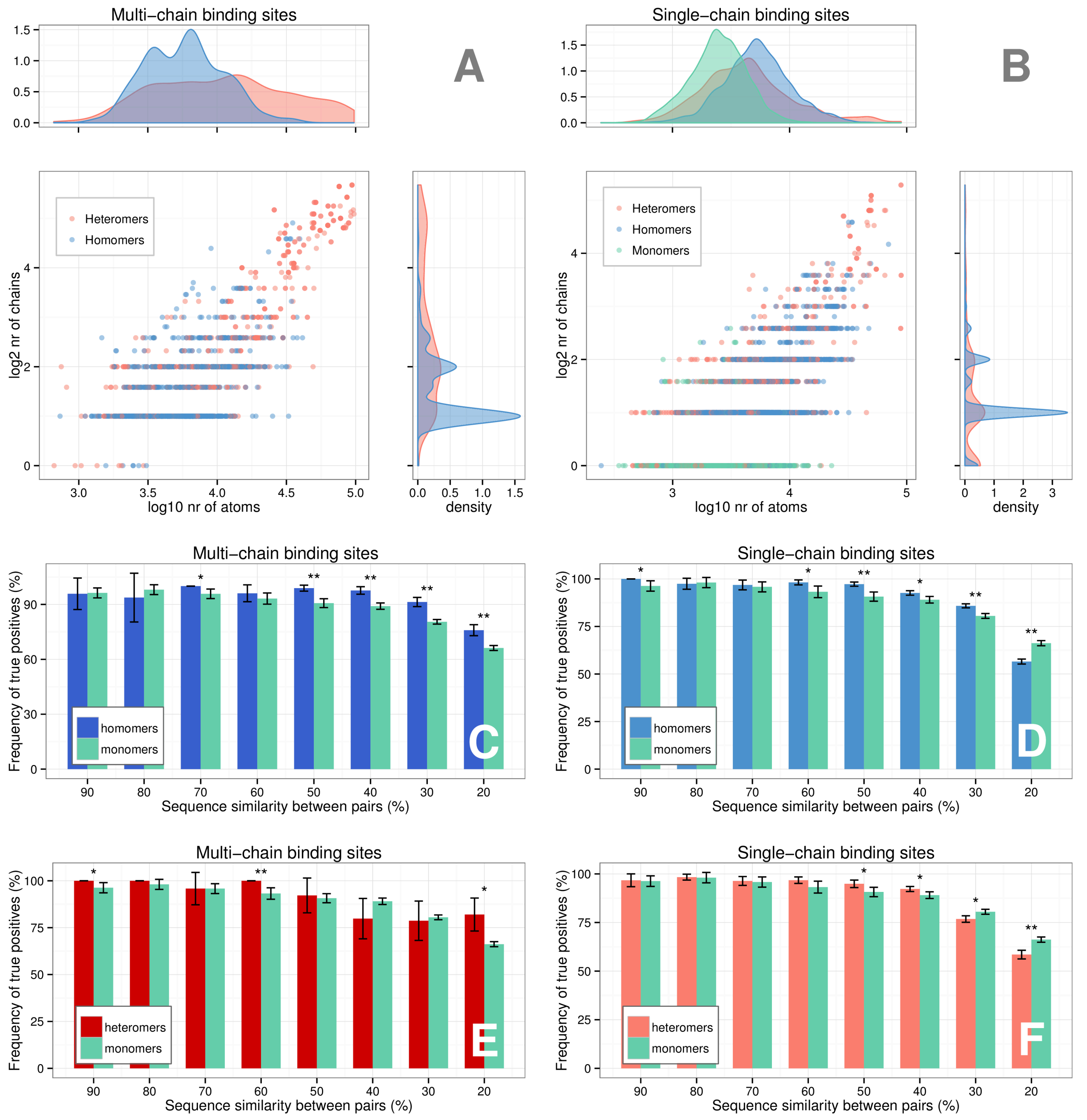

Figure S7, related to Figure 2. The efficiency of binding pocket detection is not worse in complexes than in monomers. Efficiency was determined as the fraction of significant hits between homologous structures that are known to bind the same ligand ("true positives"). **A-B)** Homomers and heteromers can have much higher numbers of chains and atoms than monomers, both in the case of complexes with multi- and single-chain binding sites. **C-F)** Despite the larger structures of complexes, the detection rate of pockets with similar ligands is comparably high in complexes and monomers, and in the case of homomers with multi-chain binding sites it is even higher than in monomers (whiskers represent 95% confidence intervals, **: $p < 0.005$, *: $p < 0.05$, t-tests, with Benjamini-Hochberg correction).
